# Organoids capture tissue-specific innate lymphoid cell development in mice and humans

**DOI:** 10.1016/j.celrep.2022.111281

**Published:** 2022-08-30

**Authors:** Geraldine M. Jowett, Emily Read, Luke B. Roberts, Diana Coman, Marta Vilà González, Tomasz Zabinski, Umar Niazi, Rita Reis, Tung-Jui Trieu, Davide Danovi, Eileen Gentleman, Ludovic Vallier, Michael A. Curtis, Graham M. Lord, Joana F. Neves

**Affiliations:** 1 School for Immunology and Microbial Sciences, King’s College London, London SE1 9RT, UK; 2 Centre for Host Microbiome Interactions, King’s College London, London SE1 9RT, UK; 3 Centre for Craniofacial and Regenerative Biology, King’s College London, London SE1 9RT, UK; 4 Centre for Gene Therapy & Regenerative Medicine, King’s College London, London SE1 9RT, UK; 5 Wellcome Trust Cell Therapies and Regenerative Medicine Ph.D. Programme, London SE1 9RT, UK; 6 Wellcome and MRC Cambridge Stem Cell Institute, Puddicombe Way, Cambridge CB2 0AW, UK; 7 Department of Surgery, University of Cambridge and NIHR Cambridge Biomedical Research Centre, Hills Road, Cambridge CB2 0QQ, UK; 8 Guy's and St. Thomas' National Health Service Foundation Trust and King's College London National Institute for Health and Care Research Biomedical Research Centre Translational Bioinformatics Platform, Guy's Hospital, London SE1 9RT, UK; 9 Faculty of Biology, Medicine and Health, University of Manchester, Oxford Road, Manchester M13 9PL, UK; 10 bit.bio, Babraham Research Campus, The Dorothy Hodgkin Building, Cambridge CB22 3FH, UK

**Keywords:** innate lymphoid cell development, epithelial organoids, stromal cells, mucosal co-cultures

## Abstract

Organoid-based models of murine and human innate lymphoid cell precursor (ILCP) maturation are presented. First, murine intestinal and pulmonary organoids are harnessed to demonstrate that the epithelial niche is sufficient to drive tissue-specific maturation of all innate lymphoid cell (ILC) groups in parallel, without requiring subset-specific cytokine supplementation. Then, more complex human induced pluripotent stem cell (hiPSC)-based gut and lung organoid models are used to demonstrate that human epithelial cells recapitulate maturation of ILC from a stringent systemic human ILCP population, but only when the organoid-associated stromal cells are depleted. These systems offer versatile and reductionist models to dissect the impact of environmental and mucosal niche cues on ILC maturation. In the future, these could provide insight into how ILC activity and development might become dysregulated in chronic inflammatory diseases.

## Highlights

• Organoids robustly capture *in situ* murine and human ILC development
• Origin of ILC maturation induces lasting tissue-specific imprint
• Human gut epithelium, not stroma, supports proliferation and maturation of human ILCP

## Graphical abstract

**Figure:**
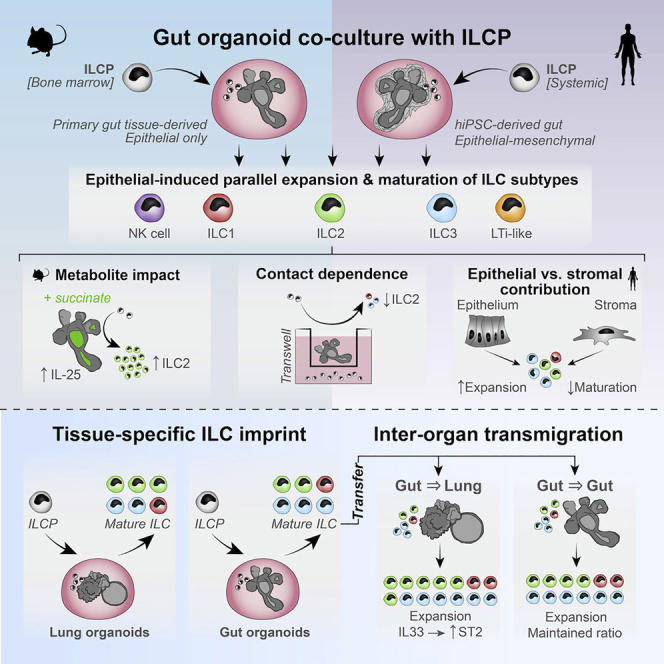

## Teaser

Jowett et al. show that intestinal and lung organoids support the generation of all innate lymphoid cells (ILCs) subsets. The phenotypes and functions of organoid-derived ILCs match *in vivo* counterparts. The epithelial niche is thus critical for the differentiation of ILCs and is sufficient to confer tissue-specific properties.

## Introduction

Innate lymphoid cells (ILCs) mediate mucosal barrier integrity during homeostasis and infection (Spits et al., 2013). Five ILC subtypes are split into three groups (Vivier et al., 2018): group 1 encompasses T-bet^+^Eomes^+^ cytotoxic natural killer (NK) cells (Kiessling et al., 1975) and T-bet^+^Eomes^−^ type 1 ILCs (ILC1s) (Klose et al., 2014), although these categories are subject to debate, especially in humans (Seillet et al., 2021; Riggan et al., 2019); group 2 consists of Rorα^+^Gata3^+^ type 2 ILCs (ILC2s) (Ferreira et al., 2021; Neill et al., 2010); and group 3 encompasses RORγt^+^CCR6^+^ lymphoid tissue inducer cells (LTis), as well as natural cytotoxicity receptor (NCR)^+/−^ type 3 ILCs (ILC3s) (Eberl et al., 2004; Mebius et al., 1997). The relative frequency and phenotypes of ILC subsets vary between tissues in mice (Dutton et al., 2018; Kim et al., 2016), and although the distinctions between subsets appear to be less rigidly defined in humans (Mjösberg and Spits, 2016), distinct ILC phenotypes are also observed between human tissues (Krämer et al., 2017; Mazzurana et al., 2021; Yudanin et al., 2019). ILC frequencies are additionally altered in acute infection, in inflammatory diseases (Bernink et al., 2013), and in cancer (Robinette et al., 2015). Understanding what drives this heterogeneity is thus a topic of considerable therapeutic interest (Cobb and Verneris, 2021).

Adult ILCs stem from the common lymphoid precursor (CLP) in the bone marrow, which differentiates toward an ILC-restricted Lineage^−^CD127^+^Id2^+^interleukin (IL)-7R^+^α4β7^+^PD-1^+^ ILC precursor (ILCP) (Constantinides et al., 2014; Mebius et al., 2001; Walker et al., 2019; Xu et al., 2019). In mice, these reportedly retain LTi-fate potential (Klose et al., 2014; Walker et al., 2019) and can also produce committed CD25^+^ ILC2 precursors (ILC2Ps) while still in the bone marrow (Yu et al., 2016). In humans, a heterogeneous systemic c-KIT^+^ ILCP population was identified in peripheral blood (Lim et al., 2017; Nagasawa et al., 2019). Recent efforts to elucidate *in situ* cues that drive distal ILC maturation have predominantly focused on mesenchymal stromal cells (Zhou and Sonnenberg, 2020), because these produce ILC survival factors, including IL-7Rα/CD127 ligand IL-7 (Sheikh and Abraham, 2019), insulin growth factor 1 (IGF-1) (Oherle et al., 2020), ST2-ligand IL-33 (Dahlgren et al., 2019), and tumor growth factor β1 (TGF-β1) (Denney et al., 2015; Puttur et al., 2019; Wang et al., 2020). However, many of these can also be derived from non-mesenchymal cells, including epithelial cells. Indeed, ILCs engage in numerous bi-directional interactions with the epithelium: ILC1 drives epithelial cell proliferation through TGF-β1 (Jowett et al., 2021); ILC2 proliferates in response to Tuft cell-derived IL-25 (von Moltke et al., 2016; Walker and McKenzie, 2013); ILC3 drives Lgr5^+^ intestinal stem cell proliferation through IL-22 (Lindemans et al., 2015; Zeng et al., 2019); and fetal LTi mediates development of secondary lymphoid structures (Mebius et al., 1997), although NK cells are circulatory and not specifically enriched in mucosa (Seillet et al., 2021; Vivier et al., 2018). This suggests that altered ILC subset ratios could impact the nature and frequency of these interactions, yet the role of epithelial cells in contributing to tissue-specific subset phenotypes has not been widely explored.

Here, we report that the epithelial niche provides critical cues for the maturation of all ILC subsets in parallel, even in the absence of other cell types or microbial tropism, and that gut and lung epithelial cells drive lasting tissue-specific ILC phenotypes.

## Results

### SIOs promote development of ILCs from ILCPs

**Figure 1:**
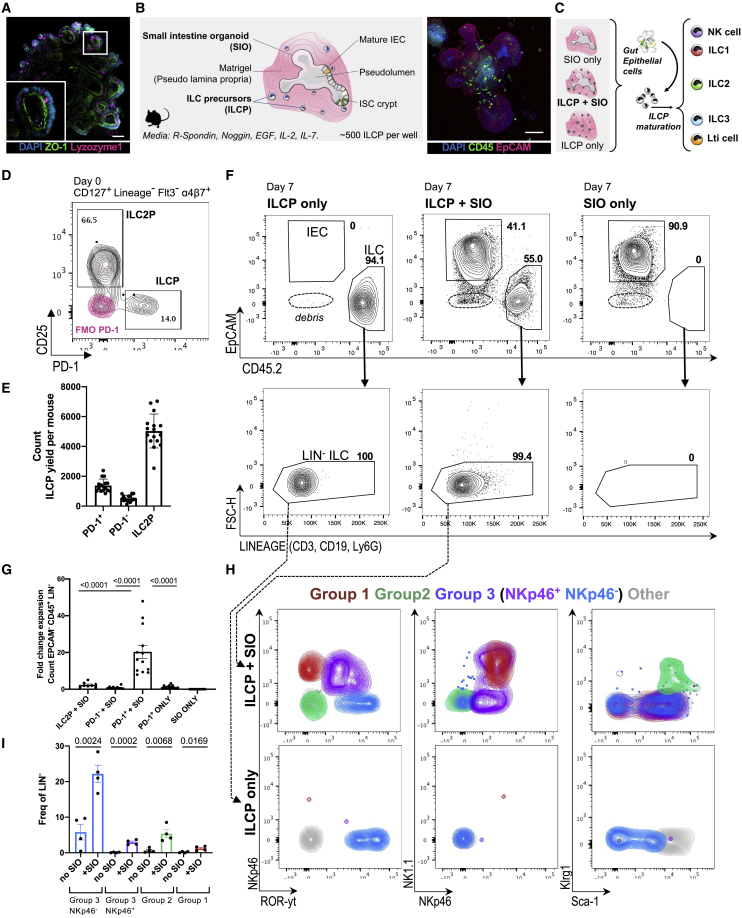
Murine ILCPs yield group 1, 2, and 3 ILCs after co-culture with SIOs (A) Confocal image of SIOs with apical ZO-1 and Lyzozyme-1^+^ Paneth cells (scale bar: 50 μm). (B) Diagram of co-culture system and representative confocal image of SIO-ILCP co-culture (scale bar: 50 μm). (C) Schematic of experimental design. (D) Representative flow plot of DAPI^−^Lineage^−^ (hematopoeitic stem cell [HSC] cocktail [CD3, CD45R, CD11b, TER-119] Ly-G6, CD5, CD19, and NK1.1), CD127^+^α4β7^+^Flt3^−^, PD-1^+^ ILCP, and CD25^+^ ILC2P, gated based on PD-1 fluorescence minus one (FMO) controls (overlaid in red). (E) Quantification of ILCP count yielded per animal (N = 15 animals, five experiments). (F) Representative flow plots of DAPI^−^EpCAM^+^CD45^−^ IECs and EpCAM^−^CD45^+^ ILCs from 7-day cultures of PD-1^+^ ILCPs only, ILCP + SIO, and SIO-only cultures. Arrows indicate corresponding lack of Lineage markers. (G) Fold change of CD45^+^Lineage^−^ cells yielded after culture relative to the number of seeded precursors (N = 15 animals, five independent experiments). (H) Contour plot overlay representing expression of NKp46, RORγt, NKp46, NK1.1, Klrg1, and Sca-1 in Lineage^−^ populations derived from (F) in populations pregated as putative group 1 (magenta; Live, EpCAM^−^, CD45^+^, Lin^−^, RORγt^−^, Klrg1^−^, NK1.1^+^, NKp46^+^), group 2 (green; Live, EpCAM^−^, CD45^+^, Lin^−^, RORγt^−^, NKp46^−^, Klrg1^+^, Sca-1^+^), NKp46^+^ group 3 (lavender; Live, EpCAM^−^, CD45^+^, Lin^−^, Klrg1^−^, RORγt^+^, NKp46^+^), NKp46^−^ group 3 (blue; Live, EpCAM^−^, CD45^+^, Lin^−^, Klrg1^−^, RORγt^+^, NKp46^−^), and a putative immature “other” population (gray; Live, EpCAM^−^, CD45^+^, Lin^−^, RORγt^−^, Klrg1^−^, NK1.1^−^, NKp46^−^) in cultures derived from ILCPs only or ILCP co-culture with SIOs. (I) Quantification of pooled ILC groups depicted in (H) (ILCPs from N = 4 animals). Error bars represent SEM; p values are from unpaired Student’s t tests.

Murine small intestine epithelial organoids (SIOs) consist of Lgr5^+^Lysozyme^+^ stem cell crypts (Figure 1A), which bud into the surrounding extracellular matrix (Barker et al., 2007; Ootani et al., 2009; Sato et al., 2009). They form tight junctions expressing zonula occludens 1 (ZO-1^+^), retaining an apico-basally polarized pseudo-lumen (Figure 1A), and are devoid of non-intestinal epithelial cells (non-IECs), such as immune or mesenchymal cells. To assess the capacity of the basal epithelium to provide a niche for ILC maturation, we harvested CD127^+^, Lineage^−^, Flt3^−^, α4β7^+^, CD25^−^, PD-1^+^ ILCPs from adult murine bone marrow and reseeded them in 3D Matrigel domes either with or without intact SIOs (Figures 1B–1E and S1A). As expected, no ILCs were observed in SIO-only cultures (Figure S1B). In SIO co-cultures, ILCPs moved freely within the Matrigel bubble and could thus access basally presented or secreted epithelial ligands (Figures S1C–S1F). After 7 days, CD45^+^, Lineage^−^ immune cells were dissociated from EpCAM^+^ IECs for downstream analysis (Figure 1F), which were identified by their expression of epithelial cellular adhesion molecule (EpCAM^+^). PD-1^+^ ILCPs significantly expanded in SIO co-culture (20.33-fold change [FC] from seeded cells) (Figure 1G). The same culture conditions in Matrigel without SIOs resulted in an 0.56-FC decrease of ILCPs. SIOs additionally promoted expansion of CD25^+^ ILC2Ps, but not control CD25^−^, PD-1^−^ immune cells (Figure 1G).

**Figure 2:**
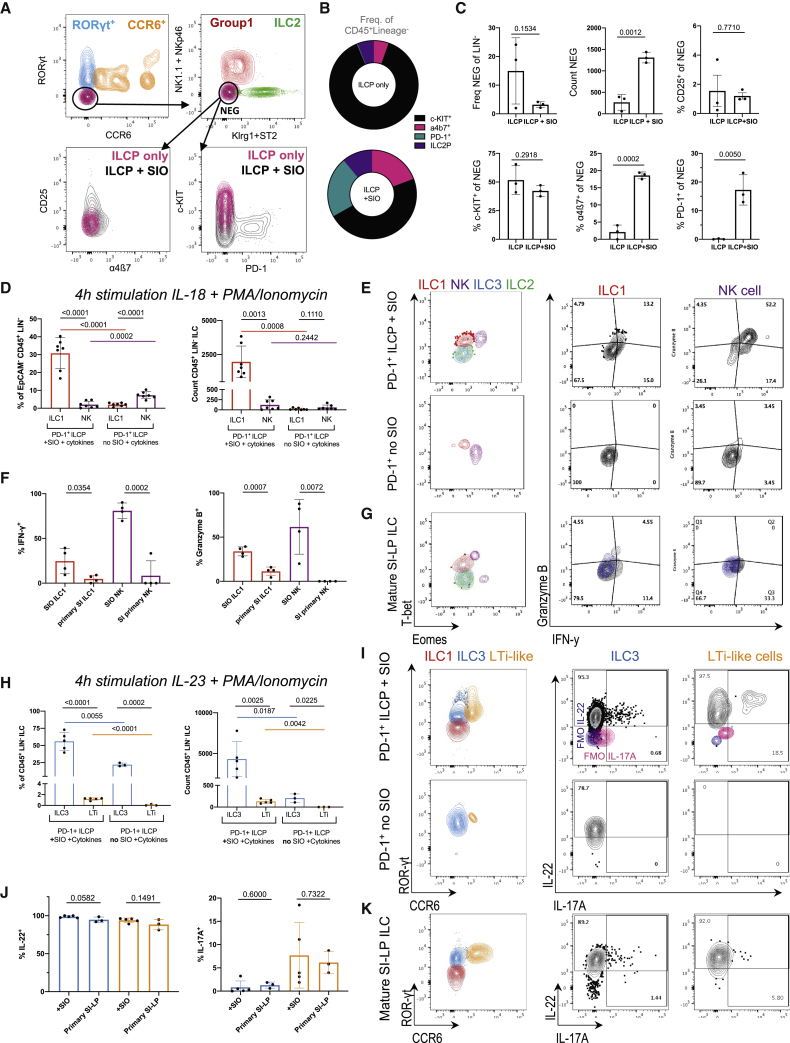
SIOs promote ILCP stemness and maturation (A) Representative flow contour plots of ILCs yielded from ILCP + SIO or ILCP only in Matrigel (“NEG” black = ILCP + SIO; “NEG” magenta = ILCP without SIO). (B) Relative frequency of α4β7^+^PD-1^+^CD25^+^c-KIT^+^ of all Lineage^−^ cells. (C) Relative frequency of RORγt^−^CCR6^−^NK1.1^−^NKp46^−^Klrg1^−^ST2^−^ (NEG) within the Lineage^−^ population, count of NEG, % of CD25^+^, c-KIT^+^, α4β7^+^, and PD-1^+^ within the negative population (frequency of parent, SD). (D) Frequency and count of ILC1 (Live, EpCAM^−^, CD45^+^, Lin^−^, RORγt^−^, Klrg1^−^, NK1.1^+^, NKp46^+^, Eomes^−^, T-bet^+^) and NK cells (Live, EpCAM^−^, CD45^+^, Lin^−^, RORγt^−^, NK1.1^+^, NKp46^+^, T-bet^+^, Eomes^+^) depicted in (E) derived from PD-1^+^ ILCP co-cultures +/− SIO and compared with putative ILC3 (Live EpCAM^−^CD45^+^Lin^−^RORγt^+^Klrg1^−^NK1.1^+/−^NKp46^+/−^Eomes^−^T-bet^+/−^) and ILC2 (Live EpCAM^−^CD45^+^Lin^−^RORγt^−^Klrg1^+^NK1.1^−^NKp46^−^Eomes^−^T-bet^−^) (N = 7 animals, two experiments). (F) Frequency of ILC1 and NK cells expressing IFN-γ (FMO, blue) in (E) and granzyme B after 4-h stimulation with PMA/Ionomycin and IL-18 compared with SI-LP ILC1s and NK cells depicted in (G). (H) Frequency and count of ILC3 (Live EpCAM^−^CD45^+^Lin^−^NK1.1^+/−^NKp46^+/−^Klrg1^−^ST2^−^RORγt^+^CCR6^−^) and CCR6^+^ LTi-like cells (Live EpCAM^−^CD45^+^Lin^−^NK1.1^−^NKp46^−^Klrg1^−^ST2^−^RORγt^+^CCR6^+^) depicted in (I) derived from PD-1^+^ ILCP co-cultures +/− SIOs and compared with primary ILC3s (N = 5 animals, two experiments). (J) Frequency of ILC3 and CCR6^+^ LTi-like cells expressing IL-22 (FMO, blue) and IL-17A (FMO, magenta) in (I) after 4-h stimulation with PMA/Ionomycin and IL-23 when compared with SI-LP ILC3 and Lti depicted in (K). Error bars represent SEM; p values are from unpaired Student’s t tests.

ILCPs were next derived from RORγt reporter mice (*Rorc*^GFP/+^) to assess whether SIO mediated maturation, as well as expansion. RORγt^**−**^ ILCPs cultured with SIOs, but not alone, significantly upregulated expression of extracellular markers associated with group 1 (RORγt^−^, NKp46^+^, NK1.1^+^), group 2 (RORγt^−^, Klrg1^+^ ILC2), and group 3 ILCs (RORγt^+^, NKp46^+/−^) (Figures 1H and 1I). A population of lymphocytes that did not express these maturation markers was also present in both cultures (Figure 1H). Thus, additional markers associated with both differentiated and immature ILC subsets were assessed (Figure 2A). The population that was negative for the expanded extracellular maturation markers (NEG) broadly expressed ILCP marker c-KIT, and there was no significant difference in the relative proportion of c-KIT^+^ and CD25^+^ cells in the NEG population with or without SIOs (Figures 2A–2C). However, SIOs significantly promoted expansion of NEG and expression of gut-homing integrin α4β7 and ILCP marker PD-1 in NEG cells (Figure 2C). This suggests that the adult gut epithelium has the capacity to actively sustain a heterogeneous population of ILCPs.

Because ILC maturation was not driven by active suppression or inability to retain ILCP stemness, the capacity of IECs to support ILC differentiation was further investigated. To assess the functional maturity of putatively mature, organoid-derived ILC groups, their capacity to mount subset-specific cytokine responses was compared with primary adult small intestine lamina propria (SI-LP)-derived ILCs. ILCPs cultured with SIOs produced significantly more Eomes^−^T-bet^+^ ILC1s relative to ILCPs cultured without SIOs (Figures 2D and 2E). A small population of T-bet^+^Eomes^+^ NK cells was also present in co-cultures (Figures 2D and 2E), in line with recent reports of shared ILC1/NK cell lineage (Walker et al., 2019; Yu et al., 2016). However, SIOs did not impact the absolute number of NK cells arising from ILCPs (Figure 2D). When stimulated with non-specific PMA and Ionomycin, ILCs from these co-cultures demonstrated the capacity to express interferon-γ (IFN-γ), even without supplementation of IL-12, IL-15, or IL-18 (Figure S2A). Moreover, an equal or greater number of ILC1s and NK cells expressed IFN-γ and granzyme B on subset-specific IL-18 stimulation in co-culture than equivalent group 1 cells isolated from primary SI-LP (Figures 2F and 2G). This suggests that SIOs drive maturation of functional group 1 ILCs. Finally, SIOs were cultured with common helper ILPs (ChiLPs) derived from T-bet^AmCyan^ reporter (Yu et al., 2015), a T-bet^YFP^ fate-mapper (Schroeder et al., 2021), and T-bet/*Tbx21*^*−/−*^ knockout lines (Figure S2B). As anticipated, Lineage^−^, Klrg1^−^, NK1.1^+^, NKp46^+^ ILCs (encompassing putative ILC1s, NK cells, and NCR^+^ ILC3s) derived from SIO co-cultures expressed T-bet^AmCyan^ and T-bet^YFP^, respectively. This same population was significantly depleted in co-cultures with *Tbx21*^−/−^ ChiLPs or with control co-cultures of *Tbx21*^+/+^CD25^−^ or PD-1^−^ ILCPs (Figures S2B and S2C). This faithfully recapitulates the lack of ILC1s observed in *Tbx21*^−/−^ animals *in vivo* (Klose et al., 2014), underscoring the versatility of this organoid approach for dissecting genetic mechanisms of ILC development.

Next, group 3 maturation was assessed. SIOs drove significant expansion of RORγt^**+**^ group 3 ILCs, including the CCR6^+^RORγt^**+**^ LTi-like population, which was exclusively observed in SIO culture (Figures 2H and 2I). This suggests that all group 3 ILC subtypes maintain a shared lineage potential from PD-1^+^ ILCPs, as was previously suggested for ChiLPs (Klose et al., 2014). After a 7-day co-culture, ∼5% of RORγt^**+**^ cells expressed IL-22 and ∼2.5% expressed IL-17A on 3-h unbiased stimulation with PMA/Ionomycin (Figure S2D). SIO-derived ILCs were additionally highly responsive to subset-specific IL-23 stimulation, secreting IL-22 at comparable rates to primary small intestine ILC3 (Figures 2J and 2K). Unlike unbiased PMA/Ionomycin, this stimulation induced some RORγt and moderate IL-22 expression even in ILCPs cultured without SIOs, although the absolute number and proportion of maturation were significantly decreased relative to ILCPs cultured with SIOs (Figure 2I). Both SIO-derived and primary ILC3 expressed low levels of IL-17A, which was more abundant in CCR6^+^ LTi-like cells (Figures 2J and 2K). Significantly fewer ILC3 and LTi-like cells were present in co-cultures with control PD-1^−^ ILCPs or CD25^+^ ILC2Ps (Figures S2E and S2F).

### SIOs recapitulate ILC subset ratios characteristic of the small intestine

**Figure 3:**
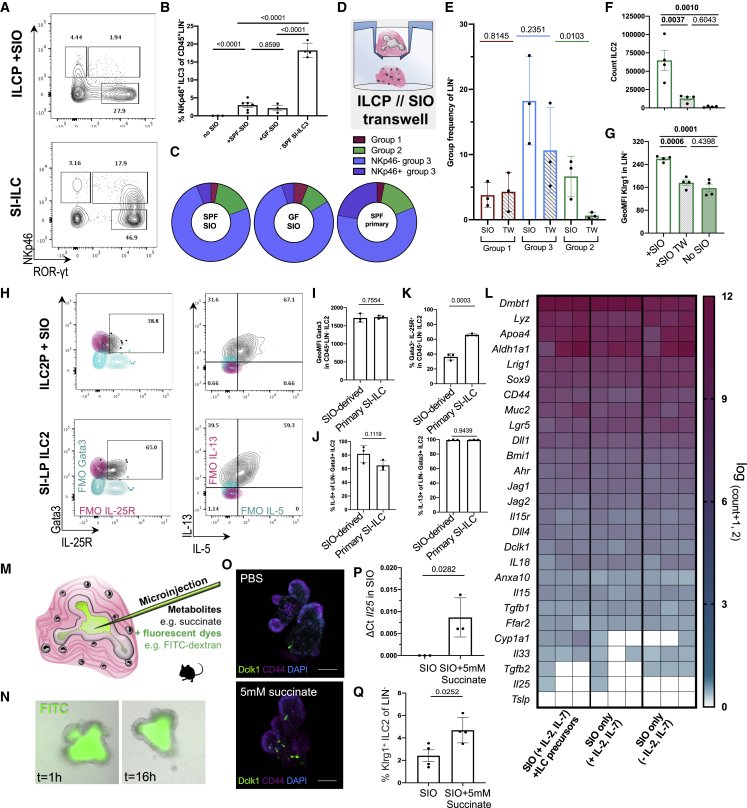
SIOs provide an essentially GF model of gut-specific ILCs (A) Representative flow plots of NKp46 expression in ILCP + SIO co-culture-derived ILCs or SI-LP-derived CD127^+^ ILCs, with the frequency of NKp46^+^ ILC3s (co-culture: Live, EpCAM^−^, Lin^−^, CD45^+^, RORγt^+^; primary tissue: Live, CD45^+^, Lin^−^, CD127^+^, Klrg1^−^, NK1.1^+/−^, RORγt^+^) additionally quantified for ILCPs cultured without SIOs or with GF SIOs in (B) (N = 2–5 animals, pooled from two experiments). (C) Relative frequency of mature ILC subsets excluding immature or other cells, depicting group 1 (magenta; Live, EpCAM^−^, CD45^+^, Lin^−^, RORγt-, ST2^−^, Klrg1^−^, NK1.1^+^, NKp46^+^), group 2 (green; Live, EpCAM^−^, CD45^+^, Lin^−^, RORγt^−^, NK1.1^−^, ST2^+^, Klrg1^+^, Sca-1^+^), NKp46^+^ group 3 (lavender; Live, EpCAM^−^, CD45^+^, Lin^−^, ST2^−^, Klrg1^−^, RORγt^+^, NKp46^+^), and NKp46^−^ group 3 ILCs (blue; Live, EpCAM^−^, CD45^+^, Lin^−^, ST2^−^, Klrg1^−^, RORγt^+^, NKp46^−^) in live, unstimulated co-cultures derived from SPF-SIOs or GF-SIOs compared with primary SPF ileum (no Peyer’s patches). (D) Diagram of transwell culture strategy. (E) Relative frequency of group 1, 2, and 3 ILCs derived from PD-1^+^ ILCP + SIO +/− transwell insert (TW) separation (N = 3, two experiments). (F and G) Count of putative LIN^−^, RORγt^−^, NKp46^−^, Klrg1^+^, Sca-1^+^ ILC2 (F) and geometric mean fluorescence intensity (GeoMFI) of Klrg1 in LIN^−^ ILCs after co-culture of CD25^+^ ILC2Ps with SIOs, TW separation, or without SIOs (N = 4, two experiments) (G). (H–K) Representative flow plots indicating expression of Gata3, IL-25R, IL-13, and IL-5 in ILC2P-derived and SI-LP-derived ILC2 after 4-h stimulation with PMA/Ionomycin (H), quantified in (I)–(K) (FMO, cyan and magenta; error bars represent SD; N = 3). (L) Gene expression heatmap (magenta = high, cyan = low, white = not detected) of genes of interest derived from bulk RNA sequencing of EpCAM^+^, CD45^−^ IECs after 7-day co-culture with precursor-derived lymphocytes, without immune cells but with IL-2, IL-7, and Flt3-ligand supplementation, or in basal SIO media. (M) Schematic of metabolite microinjection strategy. (N) SIOs microinjected with 20-kDa FITC-dextran and 5 mM succinate 16 h after injection. (O) Representative confocal images of SIOs microinjected with PBS or with 5 mM succinate stained for Tuft cell marker Dclk1 (green) and crypt marker CD44 (magenta) (scale bars, 50 μm). (P) Expression of *Il25/Il17E* normalized to *Hprt1* in SIOs injected with PBS or 5 mM succinate (n = 3 wells of SIOs in one experiment). (Q) Frequency of Klrg1^+^ ILC2s after co-culture of ILC2Ps with SIOs injected with PBS or with 5 mM succinate (ILC2Ps split between conditions from N = 4 animals in one experiment). Error bars represent SEM; p values are from unpaired Student’s t tests.

Cytokine production of SIO-derived ILC3 rivals that of primary ILC3 (Figure 2J); however, only ∼3% express NCR NKp46 compared with ∼18% NCR^+^ ILC3 in primary SI (excluding Peyer’s patches) (Figures 3A and S3). It has previously been reported that ILC expression of NCRs is in part regulated by the microbial landscape (Gury-BenAri et al., 2016). Although ILCs are present in the germ-free (GF) intestine, absence of microbiota may impact expression of epithelial Notch ligands (*DLL1*, *JAG1*, *JAG2*) that are essential, but not sufficient, for the final maturation of adult NCR^+^ ILC3s (Possot et al., 2011; Rankin et al., 2013). Intestinal organoids can maintain the epigenetic signatures of the donor tissue from which they were derived (Noben et al., 2017). However, whether serially passaged organoids resemble GF epithelium *in vitro* (Hausmann et al., 2020) or they retain epigenetic signatures of previous specific pathogen-free (SPF) microbial exposure (Janeckova et al., 2019) remains contested. To address whether SIOs were recapitulating an SPF or GF epithelial niche, we additionally derived SIOs from GF mice. Both serially passaged SPF SIOs and GF SIOs yielded predominantly NCR^−^ ILC3 from ILCPs and did not significantly differ in their capacity to drive ILC maturation, suggesting that serially passaged SPF-derived SIOs provide an essentially GF model of the epithelium (Figure 3B).

Although NKp46 expression differs between primary and SIO-derived ILC3s, the overall distribution of mature ILC groups in unstimulated SIO cultures closely resembles the ratio of ILC subsets observed in the homeostatic SI-LP, which is highly enriched for group 3 ILCs during homeostasis (Figure 3C). To evaluate whether ILCPs required contact with the epithelium to induce these intestinal ILC subset distributions, we separated ILCPs from SIOs using a transwell insert (TW) (Figure 3D). This did not affect group 1 and group 3 ILCs, but significantly reduced the number of putative ILC2s (Figure 3E). These experiments were repeated with CD25^+^ ILC2Ps, because their relative abundance within the murine bone marrow (Figure 1D) allowed for separation into more experimental conditions from the same animal. The loss of RORγt^−^Klrg1^+^ cells on TW separation was recapitulated with ILC2Ps, with the frequency of putative ILC2 (Figure 3F) and the intensity of gut-characteristic Klrg1 expression (Huang et al., 2015) (Figure 3G) both significantly decreasing when cultured either without SIOs or with SIOs separated by a TW. The maturity of ILC2P-derived ILC2 was next compared with primary ILC2. SIO co-culture and primary tissue-derived ILC2 did not differ in their expression of type 2 transcription factor Gata3 (Figures 3H and 3I), and when stimulated with PMA/ionomycin, ∼80% of ILC2s expressed IL-5, and almost all ILC2s expressed IL-13, showing no significant differences in cytokine expression relative to primary ILC2 (Figures 3H and 3J).

These data indicate that IECs have a potent capacity to support maturation of functional ILC2. However, much like NKp46, expression of IL-25R was significantly decreased in SIO-derived ILC2s relative to primary intestinal ILC2s (Figure 3K). Because ILC2s develop in anticipation of gut microbiota (Ricardo-Gonzalez et al., 2018), it was next investigated what stimuli the essentially GF SIOs might contribute to drive an IL-25R^low^ ILC2 maturation phenotype. SIOs were cultured in basal medium (SIOs only), ILC medium (with IL-2 and IL-7), and in ILC medium with ILCPs; then IECs were isolated and characterized by bulk RNA sequencing (Figure 3L). Basally presented or secreted gene targets associated with ILC maturation were assessed, including genes for expression of Notch ligands Dll1, Dll4, Jag1, and Jag2 (Golub, 2020; Possot et al., 2011; Rankin et al., 2013), cytokines IL-15 (Robinette et al., 2017) and IL-18 (Muñoz et al., 2015), and basally secreted growth factors from Bmp, Wnt, Fgf, and Tgf families (Data S1). No target genes of interest were significantly differentially expressed between culture conditions, nor were transcriptional signatures associated with small molecule biosynthesis (including genes for ILC3-associated retinoic acid [RA] synthesizing enzyme *aldh1a1*) (Goverse et al., 2016; Kim et al., 2015; Willinger, 2019). Although it is known that ILC-derived cytokines can have a significant impact on SIO gene expression (Jowett et al., 2021; Lindemans et al., 2015), these data suggest that the presence of unstimulated ILCPs does not alter the expression of these epithelial factors. Instead, these data suggest a steady-state capacity of the intestinal epithelium to support ILCP maturation, which precedes and is not dramatically altered by co-culture with ILCPs.

Next, genes specifically associated with ILC2 activity were assessed. Despite low-level expression of Tuft cell marker Doublecortin-like kinase 1 (*Dclk1*) in SIOs, expression of the Tuft cell-derived IL-25R/IL-17RB ligand *Il25* was either low or entirely absent in all three culture conditions (Figure 3L). IL-25 drives ILC2 expansion and activation (von Moltke et al., 2016) but is reportedly not required for ILC2 development (Schneider et al., 2018, 2019). This *in vivo* observation is recapitulated by the robust maturation of ILC2s from ILC2Ps in SIO co-cultures, which these data suggest occurs in the absence of SIO-derived IL-25 (Figure 2H). In adult animals, it is reported that expression of epithelial-derived cytokines is modulated by luminal metabolites. For instance, succinate contributes to IL-25/IL-17E expression in Tuft cells (Schneider et al., 2018). The absence of IL-25 in SIOs provided an optimal template to assess the capacity for these essentially GF organoids to recapitulate the well-established Succinate-Tuft-ILC2 circuit (Nadjsombati et al., 2018; Schneider et al., 2018). When microinjected into the SIO pseudolumen, fluorescein isothiocyanate dextran (FITC; 20 kDa) macromolecules are stably retained, demonstrating that epithelial barrier integrity is sufficiently maintained to create distinct inner and outer compartments separating ILCs from pseudo-luminal metabolites (Figures 3M and 3N). When injected with succinate, the number of Dclk1^+^ Tuft cells increased in SIOs (Figure 3O), correlating with increased SIO expression of *Il17e/Il25* (Figure 3P). ILCPs co-cultured with succinate-injected SIOs yielded significantly more Klrg1^+^ ILC2s (Figure 3Q). This suggests that despite low-level expression of IL-25R, SIO-derived ILC2s retain the physiological capacity to relatively expand in response to epithelial-derived IL-25. This proof of principle suggests that the impact of luminal components on *in situ* ILC development and activation in mucosal tissues can be investigated with this organoid co-culture approach.

### Epithelial identity drives tissue-specific ILC2 phenotypes

**Figure 4:**
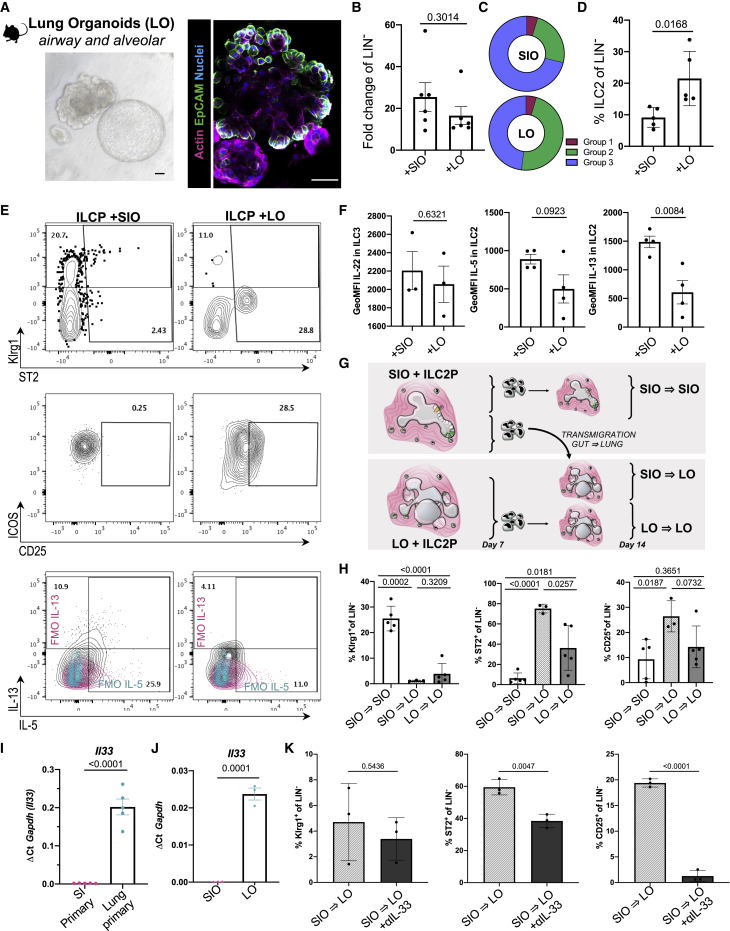
Co-culture with gut and lung organoids (LOs) drive tissue-specific ILC2 imprinting (A) Representative bright-field and confocal image of murine primary distal lung epithelial organoid showing cystic or saccular structures (scale bars: 25 μm). (B) Fold change expansion of Live, EpCAM^−^, CD45^+^ ILCPs after 7-day co-culture with SIOs or LOs (N = 6 animals, two independent experiments). (C) Relative proportion of mature ILC groups, showing Live, EpCAM^−^, CD45^+^ group 1 ILCs (magenta, Live EpCAM^−^CD45^+^Lineage^−^RORγt^−^NKp46^+^NK1.1^+^Gata3^−^), ILC2 (green, gated as Live EpCAM^−^CD45^+^Lineage^−^RORγt^−^NKp46^−^NK1.1^−^Gata3^+^), and ILC3 (blue, EpCAM^−^CD45^+^Lineage^−^RORγt^+^NKp46^+/−^NK1.1^+/−^Gata3^−^) derived from 7-day co-culture of ILCPs with SIOs or LOs. (D) Quantification of absolute ILC2 frequency in (C) as a proportion of all LIN^−^ cells (N = 5 animals). (E) Representative flow plots of Klrg1, ST2, ICOS, CD25, IL-13, and IL-5 expression in EpCAM^−^CD45^+^NKp46^−^NK1.1^−^RORγt^−^ putative ILC2s after co-culture with SIOs or with LOs (FMOs, magenta and blue), with the GeoMFI of IL-5 and IL-13 cytokine expression in ILC2 and IL-22 expression in RORγt^+^ ILC3 quantified in (F) after 4-h unbiased PMA/Ionomycin stimulation (N = 4, two experiments). (G) Schematic of SIO-to-LO experimental design. (H) Frequency of Klrg1^+^, ST2^+^, and CD25^+^ ILC2s after 14-day co-culture (N = 3–5 animals). (I) Relative gene expression of *Il33* in whole primary small intestine and lung tissue (N = 5 animals), as well as in SIOs and LOs (n = 3 wells of organoids) in (J). (K) Frequency of Klrg1^+^, ST2^+^, and CD25^+^ ILC2s in SIO-to-LO co-cultures with and without neutralizing dose of rmIL-33 blocking antibody (50 ng/mL, N = 3 animals). Error bars represent SEM; p values are from unpaired Student’s t tests.

ILC3s are abundant in the adult murine small intestine, whereas ILC2s are relatively enriched in the post-natal murine lung (Dutton et al., 2018; Saluzzo et al., 2017). To assess whether epithelial identity alone was sufficient to drive these patterns from bone marrow ILCPs, we derived murine lung organoids (LOs) from adult EpCAM^+^-enriched primary lung tip epithelial cells. These yielded a mix of cystic and saccular organoids structures (Figure 4A). Cystic organoids consist of airway epithelial cells, rich in club, goblet, and ciliated cells, whereas budding saccular structures are rich in surfactants, containing alveolar AEC1 and AEC2 cells that enable gas exchange (McQualter et al., 2010). Thus, LOs provide a rich snapshot of the many cell types of a complex lung epithelium. Much like SIOs, LOs also support expansion (Figure 4B) of putative group 1, group 2, and group 3 ILCs from common helper-like ILCPs (Figures 4C, 4D, and S4A). Notably, the relative frequency of ILC2s significantly increased in the LO co-cultures (Figure 2D).

Meta-analysis of primary murine gut and lung ILC2s (Ricardo-Gonzalez et al., 2018) provided tissue-specific ILC2 markers, such as intestinal IL-13 and pulmonary IL-33 receptor ST2 (Figure S4B). Despite an absence of other immune cells, stromal cells, or tissue-specific microbiota, epithelial-only SIO co-cultures yielded a higher proportion of Klrg1^+^ ILC2s than did LOs, whereas LO-derived ILC2s expressed higher levels of lung-enriched ST2 (Figure 4E). Finally, while ILC3 from both co-cultures did not differ significantly in their expression of IL-22 (Figure 4F), SIO-derived ILC2s expressed comparable IL-5 but significantly more IL-13 relative to LO-derived ILC2s (Figure 4F), recapitulating localized patterns observed *in vivo* (Figure S4C).

Tissue-specific ILC2 phenotypes become pertinent in murine pulmonary helminth infections, when Klrg1^high^ inflammatory ILC2s (iILC2s) (Huang et al., 2015) expand in response to IL-25, then transmigrate from the gut to the lung to clear infection (Huang et al., 2018). Indeed, only adoptive transfer of iILC2-rich gut ILCs results in successful pathogen clearance, whereas adoptive transfer of lung lymphocytes rich in natural ILC2 (nILC2) and bone marrow lymphocytes rich in ILCPs do not. The precise function and fate of post-migratory gut iILC2s in the lung remain elusive, because Klrg1^high^ ILC2s are no longer detected after resolution of infection (Huang et al., 2018). To model these post-migrational Klrg1^+^ ILC2 dynamics, we isolated and reseeded SIO-derived ILC populations with LOs (Figure 4G). After 7 additional days of co-culture, a significant downregulation of Klrg1 was observed in previously Klrg1^high^ SIO-to-LO derived ILC2s (Figure 4H), making them poorly distinguishable from Klrg1^low^ nILC2s. Conversely, ST2 and CD25 were significantly upregulated in SIO-derived ILC2 on transfer to LOs (Figure 4H). Interestingly, upregulation of ST2 was significantly higher in SIO-matured ILCs than in LO-derived ILCs that were reseeded with LOs. There is no reported literature documenting a reverse lung-to-gut ILC2 transmigration, and when LO-matured ILCs were swapped to SIOs, no significant differences in CD25, ST2, or Klrg1 expression were observed (Figure S4D).

To understand whether gut and lung epithelium differed in expression of ILC-associated target genes (as in Figure 3L), we performed a meta-analysis of publicly available (GEO: GSE112991) and experimentally comparable primary adult human gut and LO gene expression (Heo et al., 2018) (Figure S4E). In this dataset, lung and gut epithelial cells clustered separately, recapitulating anticipated tissue-specific expression of intestinal antimicrobial peptides (e.g., *LYZ*, *REG4*) and pulmonary surfactant proteins (e.g., *SCGB1A1*). As in the murine organoids, *IL17E/IL25* was not detected in either organoids, and the majority of basally presented or secreted genes of interest were not significantly differentially regulated. However, expression of the ST2-ligand IL-33 was significantly enriched in pulmonary epithelial cells (Figure S4F). This differential expression was conserved in primary murine small intestine and lung tissue (Figure 4I), as well as in the SIO and LO cultures (Figure 4J). A neutralizing dose of murine anti-IL-33 antibody was supplemented to co-cultures on swapping from SIOs to LOs to assess the role of tissue-specific differences in IL-33 in the phenotypic transformation of SIO-to-LO ILC2s. Inhibition of IL-33 had no impact on Klrg1 downregulation of SIO-to-LO ILC2s; however, it significantly dampened the upregulation of ST2 and CD25 (Figure 4K). To ensure that these changes were actively induced by pulmonary epithelial cells, including IL-33, and not merely resulting from a loss of intestinal-specific repressive stimuli, we additionally reseeded SIO-derived ILCs in Matrigel without SIOs or LOs, but with or without recombinant murine IL-33 (rmIL-33; Figure S4G). A loss of contact with SIOs was not sufficient to recapitulate the SIO-to-LO phenotypes, suggesting that ST2 and CD25 expression are not repressed by an intestinal milieu. Instead, recombinant IL-33 alone was sufficient to induce high levels of ST2 and CD25 expression in SIO-matured ILCs (Figure S4G). This suggests that ILC2s may acquire a lasting imprint of intestinal origin, making them more receptive to pulmonary stimuli like IL-33 than lung-derived nILC2s and offering a potential explanation for their heightened capacity to clear pulmonary helminth infections.

### Human epithelial cells, not mesenchyme, drive robust ILC expansion and maturation

**Figure 5:**
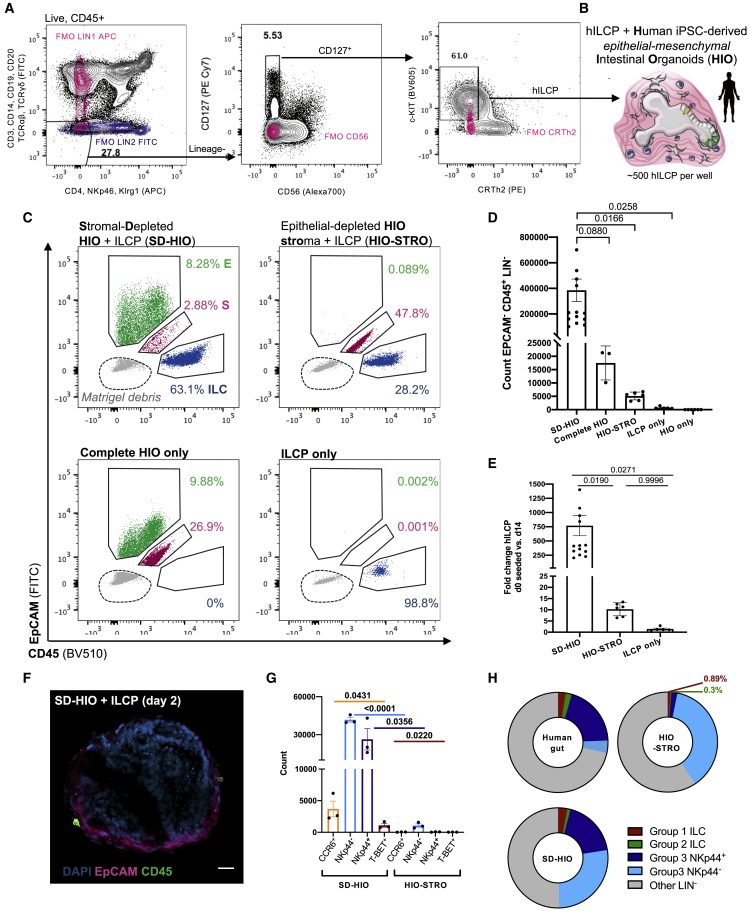
HIOs promote proliferation and maturation of systemic human ILCPs (A) Gating strategy for PBMC-derived ILCPs, pregated on live, single, CD45^+^ cells (FMOs, blue and magenta). (B) Schematic of HIO + ILCP co-cultures, indicating the presence of mesenchymal cells and CD45^+^ ILCPs. (C) Representative plot overlays of EpCAM^+^ IECs, double-negative mesenchymal cells (M, magenta), and CD45^+^ ILCs (blue) after 14-day co-cultures, highlighting Matrigel debris in gray, and quantified in (D) as count of EpCAM^−^, CD45^+^, LINn^−^ ILCs and (E) fold change expansion of ILCs after 14-day co-culture relative to the number of ILCPs seeded on day 1 (N = 3–15 across seven experiments). (F) Representative image of CD45^+^ ILCPs co-cultured with mesenchyme-depleted, EpCAM^+^ HIOs (scale bar: 25 μm). Error bars represent SEM; p values are from unpaired Student’s t tests. (G) Count of Live, EpCAM^−^CD45^+^LIN^−^RORγt^+^ ILCs after 14-day co-culture with SD-HIOs or epithelial-depleted HIO-STROs expressing markers CCR6, NKp44, and/or T-bet (ILCPs from N = 3 PBMC donors). (H) Relative group 1 (Live, CD45^+^Lin^−^CRTH2^−^c-kit^−^ [CD127^+^CD161^+^ in primary gut ILC only] and [EpCAM^−^RORγt^−^GATA3^−^ in SD-HIO and HIO-STRO only]), group 2 (Live, CD45^+^Lin^−^CRTH2^+^c-kit^+/−^, [CD127^+^CD161^+/−^ in primary gut ILC only] and [EpCAM^−^RORγt^−^GATA3^+^ in SD-HIO and HIO-STRO only]), group 3 (Live, CD45^+^Lin^−^CRTH2^−^c-kit^+^, NKp44^+/−^ and [CD127^+^ CD161^+^ in primary gut ILC only] and [EpCAM^−^RORγt^+^GATA3^−^ in SD-HIO and HIO-STRO only]), and other LIN^−^ ILCs (e.g., undifferentiated precursors; Live, CD45^+^, Lin^−^, CRTH2^−^, c-kit^−^ and [CD127^+^ CD161^+^ in primary gut ILC only] and [EpCAM^−^, RORγt^−^, GATA3^−^ in SD-HIO and HIO-STRO only]) in unstimulated, primary human intestine (N = 13, adapted from Krämer et al., 2017), SD-HIOs (N = 13 from six experiments), and HIO-STROs (N = 7 from three experiments).

Biomedical translation of murine mucosal immunology to human settings often faces technical hurdles. In this case, human bone marrow ILCPs are poorly accessible, and human patient-derived primary epithelial-only organoids risk introducing significant variability from donor genetics, age, and potential disease status. Thus, to adapt the murine ILCP-organoid system to a human model, readily available systemic ILCPs were instead isolated from human peripheral blood mononuclear cells (PBMCs) (Lim et al., 2017; Nagasawa et al., 2019). Specifically, a highly stringent Lin^−^CD127^+^NKp46^−^CD56^−^Klrg1^−^CRTh2^−^c-KIT^+^ population (Figure 5A) was selected to closely match the stringent PD-1^+^ ILCPs used in the murine system. Moreover, human intestinal organoids (HIOs) were derived from healthy human induced pluripotent stem cells (hiPSCs) (McCracken et al., 2011; Spence et al., 2011; Tsai et al., 2017). These GF structures are well established, can be matured through addition of IL-2 (Jung et al., 2018), and express the previously discussed ligands and growth factors of interest for ILCP maturation (Yu et al., 2021).

Co-culture of PBMC ILCPs with HIOs (Figures S5A and 5B) resulted in significant immune expansion (∼20-fold) relative to the number of cells seeded without HIOs and when compared with the number of ILCPs seeded on day 1 (d1) (Figure S5B). Human ILCPs developed as putative group 2 (Figure S5C), group 3 (Figure S5D), and group 1 ILCs (Figure S5E) when cultured with HIOs. A population of RORγt^+^ ILCs significantly upregulated NKp44 (Figures S5F and S5G), recapitulating the difference in c-KIT^+^ ILCs observed between peripheral and gut c-KIT^+^ ILCs *in vivo* (Lim et al., 2017). However, HIO-derived NKp44^+/−^CCR6^+/−^ group 3 RORγt^+^ ILCs produced moderate levels of IL-22 and IL-17A on non-specific PMA/Ionomycin stimulation and modestly responded to additional 4-h stimulation with IL-23 (Figure S5H), while putative group 1 ILCs upregulated expression of T-bet and IFN-γ secretion in response to 4-h stimulation with IL-18 (Figure S5E).

**Figure 6:**
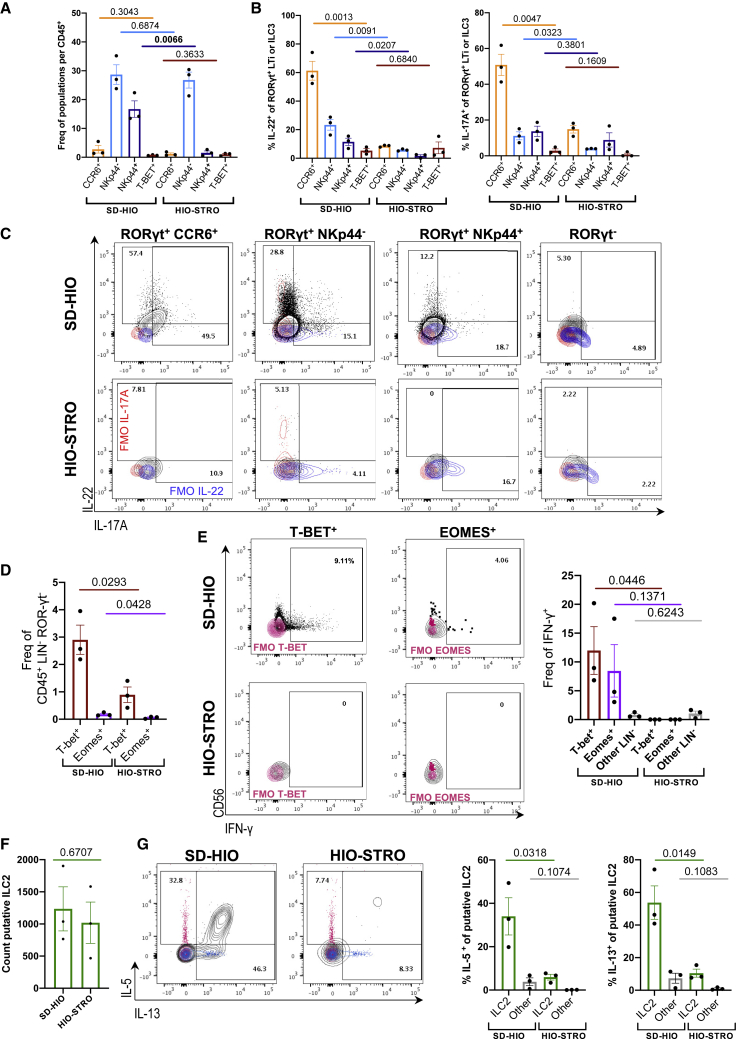
Human epithelial cells, not mesenchymal cells, drive proliferation and maturation of functional human ILCs (A) Frequency of Live, EpCAM^−^, CD45^+^, LIN^−^, RORγt^+^ ILCs after 14-day co-culture expressing markers CCR6, NKp44, and/or T-bet (ILCPs from N = 3 donors). (B) Frequency of Live, EpCAM^−^, CD45^+^, Lineage^−^, RORγt^+^ cells expressing IL-22^+^ and IL-17A^+^ in SD-HIO- and HIO-STRO-derived ILCs after 4-h stimulation with PMA/Ionomycin (ILCPs from N = 3 donors). (C) Representative flow plots corresponding to (B) (FMOs, blue and magenta). (D) Frequency of Live, EpCAM^−^, CD45^+^, LIN^−^, RORγt^−^ cells expressing T-bet and/or Eomes (ILCPs from N = 3 donors). (E) Representative flow plots and quantification of CD56 and IFN-γ expression in T-bet^+^ and Eomes^+^ populations after PMA/Ionomycin 4-h stimulation (IFN-γ FMO overlaid, magenta). (F) Total count of putative ILC2 (Live, EpCAM^−^, CD45^+^, Lineage^−^, RORγt^−^, GATA3^+^, expressing either Klrg1, CD25, ST2, and/or CRTh2) after 14-day co-culture. (G) Expression of IL-5 and IL-13 in putative ILC2s after 4-h PMA/Ionomycin stimulation (FMOs, magenta and blue). Error bars represent SEM; p values are from unpaired Student’s t tests. All experiments were performed with ILCPs from N = 3 donors.

These data suggest the HIO microenvironment may provide sufficient stimuli for maturation of human ILCPs, although less efficiently than murine SIOs. However, unlike those primary epithelial-only organoids, hiPSC-derived hindgut organoids co-develop with rich and complex native mesenchyme (Spence et al., 2011). These stromal cells not only contribute to the maturation of IECs (Stallmach et al., 1989) but offer a source for ILC survival factors like IL-7 (Xu et al., 2015). To assess whether IECs or mesenchymal cells were predominantly driving human ILCP maturation, we digested the matrix of HIO structures with Collagenase, and a single-cell suspension of stromal mesenchyme was separated from intact 3D epithelial structures through serial gravity-gradient separation, resulting in a stromal-depleted HIO fraction (SD-HIO) and an epithelial-depleted and fibroblast-rich stromal fraction (HIO-STRO) (Figure 5C). Both SD-HIO and HIO-STRO cultures maintained ILC viability and expansion from ILCPs for 14 days (Figure 5C). However, the SD-HIO condition somewhat unexpectedly yielded a dramatic increase in EpCAM^−^, CD45^+^, Lineage^−^ immune cells after culture (Figure 5D), significantly increasing the FC expansion relative to ILCPs seeded with HIO-STROs or in Matrigel only (Figure 5E). Moreover, although complete HIOs induced NKp44 expression in ∼2%–6% of group 3 ILCs (Figure S5G), SD-HIO co-cultures yielded 15%–22% NKp44^+^ ILCs (Figures 5G and 6A). Thus, SD-HIOs, but not HIO-STROs, promoted patterns of ILC subset maturation that closely resembled the distribution of mature ILCs in the healthy human intestine (Krämer et al., 2017) (Figure 5H). As in the murine model, ILCPs could freely interact with the intestinal epithelium in the SD-HIO cultures (Figure 5F), where ILCPs predominantly yielded more NCR^−^ ILC3s than would be expected from an NCR-rich primary intestine exposed to microbiota (Figures 5G and 5H). This suggests that HIOs could provide a versatile and GF model for investigating human host-microbial impacts on mucosal ILC populations.

The current gold standard for investigating ILC maturation *in vitro* relies on modified murine bone marrow stromal feeder cells (Nakano, 1996). Therefore, the relatively poor ILCP expansion rates induced by HIO-STROs were somewhat unexpected. We hypothesized that the increased cell number in SD-HIO cultures could represent proliferation of immature ILCPs, while stromal cells may yield fewer but more mature ILCs. To test this hypothesis, unbiased stimulation with PMA/Ionomycin was performed in ILCs derived from SD-HIO and HIO-STRO co-cultures, respectively. The relative frequency of CCR6^+^, NKp44^−^, and T-bet^+^ ILCs remained comparable in both conditions, yet the frequency of NKp44^+^ cells of RORγt^+^ group 3 ILCs significantly expanded with SD-HIOs (Figure 6A). Moreover, CCR6^+^ and NKp44^+/−^ ILCs expressed significantly greater amounts of IL-22, and CCR6^+^ and NKp44^−^ ILCs expressed significantly more IL-17A than HIO-STRO-derived ILCs (Figures 6B and 6C). The frequency of IL-22^+^ ILCs was either comparable or greater in unbiased SD-HIO cultures than in complete HIO cultures stimulated with IL-23 (Figure S5H). The proportion of putative group 1 CD56^+/−^, T-bet^+^, and Eomes^+^ ILCs (Figure S6A) was not only significantly decreased in HIO-STROs (Figure 6D), but no expression of IFN-γ was observed in either population in the absence of epithelial cells when stimulated with PMA/Ionomycin (Figure 6E). The proportion of T-bet^+^ ILCs and the number of IFN-γ^+^ cells was greater in SD-HIOs than in any other condition (Figures 6E and S5E).

Unlike group 1 and 3 ILCs, the overall count (Figure 6F) and relative frequency (Figure S6A) of putative group 2 ILCs (Figure S6B) were not significantly affected by the depletion of stroma. Nevertheless, expression of IL-5 and IL-13 were both significantly and greatly increased in SD-HIO fractions on unbiased stimulation with PMA/Ionomycin (Figure 6G). These data stand in contrast with murine ILC2P co-cultures, which suggested epithelial contact-dependent maturation mechanisms (Figures 3D–3F). To assess how much this may be conserved in the human system, we separated ILCPs from SD-HIOs using TW (Figure S6D). Although the yield of ILC2s was still greater in TW-separated co-cultures than in ILCPs cultured in Matrigel only, both the overall cell count and relative ILC2 frequency were decreased in TW-separated cultures, and these ILC2s failed to express IL-5 or IL-13 (Figure S6D), suggesting that final stages of ILC2 maturation or activation may have conserved cell-contact-regulated processes in humans.

Finally, we aimed to assess whether the lack of cytokine expression in HIO-STRO co-cultures was due to failed maturation, competitive depletion of activating ligands, or active inhibition of ILC2 maturation. To test this, we reseeded SD-HIO-matured ILCs either with SD-HIOs or swapped to HIO-STRO co-culture (Figure S6E). Although ILCs reseeded with SD-HIOs continued to proliferate, those transferred to epithelial-depleted cultures ceased to expand and significantly decreased expression of IL-5, although not of IL-13 (Figure S6F). This suggests that stable development of IL-13^+^ ILC2s may be promoted by intestinal epithelial contact, but ILC2 proliferation and expression of IL-5 may either be actively repressed by intestinal mesenchyme or require constant epithelial exposure.

### Human mucosal epithelial identity contributes to tissue-specific ILC maturation

ILC subset distributions are reportedly less variable between human organs than in mice (Yudanin et al., 2019), although ILCs still acquire distinct transcriptional imprints across tissues (Mazzurana et al., 2021). It also has not been possible to study putative gut-to-lung transmigration of human ILC2s, because experimental methods like adoptive transfers do not lend themselves to investigation of human biology.

**Figure 7:**
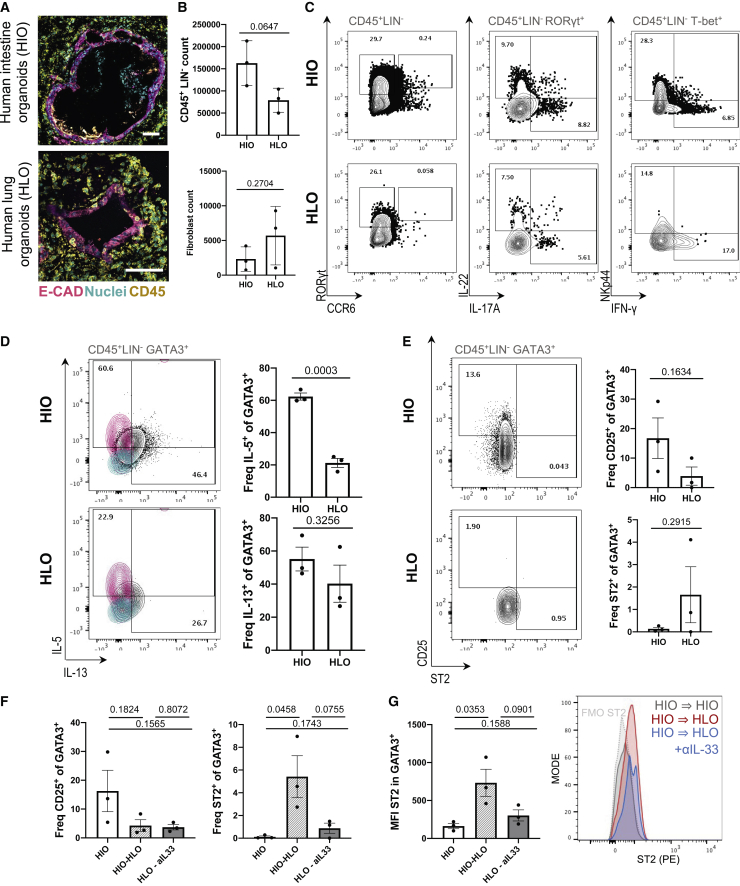
Gut-matured ILC2 upregulates ST2 on transfer to HLO culture (A) Representative image of SD-HIOs and SD-HLOs showing E-cadherin^+^ (E-CAD) epithelium, CD45^+^ ILCs, and nuclei (Hoechst) after 14-day co-culture (scale bars: 50 μm). (B) Count of EpCAM^−^, CD45^+^, LIN^−^ ILCs after 14-day co-culture with SD-HIOs or SD-HLOs, with corresponding count of EpCAM^−^, CD45^−^ mesenchyme. (C) Representative flow plots of RORγt, CCR6, IL-22, IL-17A, IFN-γ, and NKp44 after 14-day co-culture with SD-HIOs or SD-HLOs after 4-h PMA/Ionomycin stimulation (pregated population in gray, representative of N = 3). (D) IL-5 and IL-13 expression in putative GATA3^+^ ILC2s after 14-day co-culture and 4-h PMA/Ionomycin stimulation (FMOs, magenta and cyan). (E) Expression of CD25 and ST2 in putative GATA3^+^ ILC2s after 14-day co-culture with SD-HIOs or SD-HLOs. (F and G) Relative frequency of CD25^+^ and ST2^+^ putative ILC2s (F) and GeoMFI of ST2 in the same population with corresponding histogram overlay of ST2 (PE) after 14-day co-culture with SD-HIOs or SD-HLOs, followed SD-HIO-to-HIO, SD-HIO-to-HLO, or SD-HIO-to-HLO with 50 ng/mL hIL-33 neutralizing antibody (G). All experiments were performed with ILCPs from N = 3 donors; unpaired two-tailed Student’s t tests were used; and error bars represent SEM.

To assess whether any tissue-characteristic ILC phenotypes could be captured in human organoid systems (Figure 7A), we cultured ILCPs with either SD-HIOs or hiPSC-derived LOs (HLOs) (de Carvalho et al., 2019; Miller et al., 2019). ILCPs expanded in both stromal-depleted co-cultures (Figure 7B). HLOs demonstrated comparable capacity to yield mature group 1 and group 3 cells that expressed IL-22, IL-17A, and IFN-γ (Figure 7C). Both conditions yielded GATA3^+^ ILCs that expressed IL-5 and IL-13 (Figure 7D). However, much like HIO-STROs, HLOs did not support maturation of IL-5^+^ as significantly as HIO. Similarly, a recently described c-KIT^+^, CRTh2^low^, IL-17A^+^ ILC2 (Bernink et al., 2019, Hochdörfer et al., 2019) population was present in SD-HIO, but not in HLO cultures (Figure S7). One interpretation of this observation is that much like the murine system, the human intestinal microenvironment may favor group 3 maturation to the extent of inducing ex-ILC2 to ILC3 plasticity in mature SD-HIO-derived ILC2s. However, unlike in the murine system, co-culture with HIO or HLO alone was not sufficient to induce statistically significant differences in the frequency of CD25 and ST2 ILCs within this GATA3^+^ putative ILC2 population (Figure 7E).

Finally, we assessed the capacity of the human organoids to recapitulate gut-to-lung post-migrational dynamics harnessing the approach used in murine co-cultures (Figure 4G). SD-HIO-derived ILCs were reseeded with SD-HIOs, SD-HLOs, or SD-HLOs with human IL-33 antibody neutralization, as performed in the murine system. Exposure to SD-HLO increased ILC2 expression of ST2, but not CD25 (Figures 7F and 7G) (Mjösberg et al., 2011; von Moltke et al., 2016; Ricardo-Gonzalez et al., 2018). As in the murine system, this effect was dampened through addition of a neutralizing dose of IL-33-blocking antibody (Figures 7F and 7G). This suggests that the epithelial tissue origin of ILC development may impact ILC imprints in both mice and humans.

## Discussion

Organoids offer exquisite experimental control over host genetics and environment, making them an appealing choice for studying the behavior and development of mucosal immune cells in distal tissues. Here, organoids are harnessed to reveal a critical role for the epithelial niche in mediating ILC maturation. Gut and lung epithelial cells recapitulate tissue-specific ILC subset frequencies respectively, even in the absence of microbiota or other cell types. This fold expansion and development occurred without requiring the addition of IL-1β or subset-specific cytokines, whereas previous *in vitro* systems required supplementation with IL-1β+IL-23 for ILC3s, IL-25+IL-33 for ILC2s, and IL-12+IL-18 for robust ILC1 development (Lim et al., 2017), or of IL-15 and differential Notch ligands when matured from CD34^+^ hematopoietic stem cells (Hernández et al., 2021). Our findings build on existing literature linking pulmonary IL-33 to ILC2 activity (Ferreira et al., 2021; Saluzzo et al., 2017), additionally suggesting that an intestinal origin of ILC2 maturation may leave a lasting imprint, making them more susceptible to this pulmonary IL-33. It remains an outstanding question if and how additional epithelial-derived cues like TGF-β1 (Wang et al., 2020) contribute to tissue-specific ILC signatures, yet this reductionist organoid co-culture model lends itself to investigating these exciting future directions.

The observation that the HIO-associated stromal fraction alone fails to promote ILCP proliferation or cytokine expression raises important questions. The impact of epithelium-mesenchyme ratios on ILC activity underscores the significance of assessing the stromal compartment across different stages of development and disease (Buechler et al., 2021; Korsunsky et al., 2021; Elmentaite et al., 2021). Dysregulation of this mucosal niche would drive aberrant ILC accumulation and cytokine secretion based on predictions from human co-cultures with ILCPs. This is also an important consideration for *in vitro* models of ILC, either for research or cell therapeutic expansion purposes. The bone marrow niche recapitulated by OP9 feeder-based systems might logically promote immune precursor stemness and inhibit differentiation, limiting their capacity to capture physiological ILC behavior in distal mucosa. Notably, previous reports of OP9 co-cultures with the NKp46^−^ CD56^−^ Klrg1^−^CRTH2^−^c-KIT^+^ ILCPs used in this study predicted poor precursor potential of this unbiased population (Nagasawa et al., 2019). Conversely, in SD-HIO co-culture, this stringent ILCP population demonstrated a robust capacity to give rise to all ILCs in parallel. Indeed, this GF system is adept at supporting maturation of NKp46l^ow/−^ group 3 ILCs and would thus be amenable to studying immune-mediated human fetal organogenesis (Jowett et al., 2022), for instance, Peyer’s Patch development in fetal gut organoid co-cultures with Lti cells. As shown with the succinate microinjections, the system could even incorporate more complex microbial compositions to capture how microbial seeding impacts the evolving ILC landscape after birth.

### Limitations of the study

This *in vitro* system is prone to variability in differentiation efficiency. Sufficient organoids should be used to minimize this; these should be handled, differentiated, and passaged consistently prior to seeding co-cultures, and the ratio of ILCPs to organoids should be kept constant between experiments. Although this study suggests an important role for epithelial cells in driving ILC maturation, it cannot confirm that additional complex interactions with mucosal or endothelial cells *in vivo* contribute to or compete with the epithelial-ILC interactions presented in this manuscript.

## STAR★Methods

### Key resources table

**Table:** 

REAGENT or RESOURCE	SOURCE	IDENTIFIER
**Antibodies**		

Mouse Lineage cocktail – PacBlue	Thermofisher (eBioscience)	Cat# 88-7772-72
Mouse CD3 – PacBlue	Thermofisher (eBioscience)	Cat# 48-0031-82
Mouse CD19 – PacBlue	Thermofisher (eBioscience)	Cat# 48-0193-82
Mouse Ly6g – PacBlue	Thermofisher (eBioscience)	Cat# 48-5931-82
Mouse CD5 – PacBlue	Thermofisher (eBioscience)	Cat# 48-0051-82
Mouse NK1.1 – PacBlue	Thermofisher (eBioscience)	Cat# 48-5941-82 9
Mouse NK1.1– PE	Thermofisher (eBioscience)	Cat# 12-5941-83
Mouse CD127 – APC	Thermofisher (eBioscience)	Cat# 17-1271-82
Mouse a4b7 – PE	Thermofisher (eBioscience)	Cat# 12-5941-83
Mouse Flt3 – PerCP Cy.5.	Thermofisher (eBioscience)	Cat# 46-1351-82
Mouse NK1.1 – bv605	BioLegend	Cat# 108753
Mouse NKp46 – bv605	BioLegend	Cat# 137619
Mouse Klrg1 –PerCP Cy5.5	Thermofisher (eBioscience)	Cat# 46-5893-82
Mouse NKp46 – PE Cy7	Thermofisher (eBioscience)	Cat# 25-3351-82
Mouse CD45 – bv510	BioLegend	Cat# 103137
Mouse Sca-1 bv711	BioLegend	Cat# 108131
Mouse Sca1 – Alexa700	Thermofisher (eBioscience)	Cat# 56-0451-82
Mouse EpCAM – APC Cy7	BioLegend	Cat# 118217
Mouse CD25 – APC e780	Thermofisher (eBioscience)	Cat# 47-0251-82
Tbet – BV711	BioLegend	Cat# 644819
Mouse IL-22 – PE	Thermofisher (eBioscience)	Cat# 12-7221-82
Mouse IFN-γ – APC	eBioscience	Cat# 17-7311-81
Mouse IL-5 – APC	BD biosciences	Cat# 562048
Mouse IL-13 – PE Cy7	Thermofisher (eBioscience)	Cat# 25-7133-80
Mouse IL17a – PE/Dazzle	BioLegend	Cat# 506937
Mouse IL17a – APC	Invitrogen	Cat# 17-7177-81
Mouse Gata3 – AF488	BD Pharmingen	Cat# 560163
Mouse St2 – PerCPe710	Thermofisher (eBioscience)	Cat# 46-9335-82
Mouse RORγt – BV785	BD biosciences	Cat# 564723
Mouse ICOS – APC	Thermofisher (eBioscience)	Cat# 17-9949-82
Mouse CD25 - bv786	Thermofisher (eBioscience)	Cat# 14-0251-86
Human CD45 – e450, Alexa700	Invitrogen	Cat# 48-0459-42
Human EpCAM – FITC	BioLegend	Cat# 324203
Human LIN cocktail 3 – FITC/PacBlue	BD biosciences	Cat# 643510
Human CD4 – APC	Thermofisher (eBioscience)	Cat# 17-0048-42
Human KLRG1 – APC	BioLegend	Cat# 138411
Human CD335 NKp46 – APC	BioLegend	Cat# 331917
Human TCR α/β- FITC	BioLegend	Cat# 306706
Human TCR γ/δ- FITC	BioLegend	Cat# 331208
Human CD127 – PE Cy7	Thermofisher (eBioscience)	Cat# 25-1278-42
Human CD56 – Alexa 700	BioLegend	Cat# 318316
Human c-KIT/CD117 bv605	BioLegend	Cat# 313218
Human CRTh2 – PE	Miltenyi Biotec	Cat# 130-113-600
Human CRTh2– BV711	BioLegend	Cat# 350124
Human CRTh2 – BV421	BioLegend	Cat# 350112
Human CD161 APC, A700	BioLegend	Cat# 302012
Human ST2 – APC	R&D Sys	Cat# FAB5231A
Human ST2 – PE	R&D Sys	Cat# FAB5231P
Human NKp44 – PE Cy7	BioLegend	Cat# 325116
Human NKp44 – PerCP Cy5.5	BioLegend	Cat# 325114
Human/Mouse Tbet – PE-Cy7	BioLegend	Cat# 644824
Human RORγt – APC	Thermofisher (eBioscience)	Cat# 17-6988-82
Human RORγt – PE	BDBiosciences	Cat# 563081
Human GATA3 – APC Cy7	Santa Cruz	Cat# sc-268
Human IL22 – PerCP Cy5.5	BioLegend	Cat# 366709
Human IL17A – PE-Dazzle	BioLegend	Cat# 512335
Human IL17A – e450	BD Horizon/ bioscience	Cat# 560610
Human IL17A – BV786	BD Horizon/ bioscience	Cat# 563745
Human IFNg –APCe780	Invitrogen	Cat# 47-7319-41
Human IL-5 – APC	BioLegend	Cat# 504305
Human IL-13 – FITC	eBioscience	Cat# 11-7139-41
Human IL-13 – bv711	BD Biosciences	Cat# 564288
Human CD25 – PerCP-Cy 5.5	BD Biosciences	Cat# 560503
Human Klrg1 – APC	Thermofisher (eBioscience)	Cat# 25-5893-80
Human CCR6 – APC	BioLegend	Cat# 353416
Human CCR6 – BV605	BioLegend	Cat# 353419
Human NKp46 APC	BioLegend	Cat# 331918
FcR CD16/32 blocking, mouse	BioCell	Cat# BE0307
FcR blocking, human	Miltenyi Biotec	Cat# 130-059-901
Anti-rhIL33 (neutralising, ICC, goat polyclonal)	R&D	Cat# AF3625
Anti-rmIL33 (neutralising, ICC, goat polyclonal)	R&D	Cat# AF3626
E-Cadherin – anti-human (rat)	Thermofisher (eBioscience)	Cat# 51-3249-82
CDX2 – anti-human (rabbit)	abcam	Cat# Ab76541
EpCAM – anti-mouse (rabbit)	abcam	Cat# Ab71916
CD45 anti-human (mouse)	BioLegend	Cat# 304001
ZO-1 anti-mouse (rabbit)	Abcam	Cat# Ab96587
Dclk1 anti-mouse (rabbit)	Abcam	Cat# Ab31704
CD44 anti-mouse/human (rat)	Thermofisher (eBioscience)	Cat# 14-0551-82

**Critical commercial assays**		

CellTrace FarRed	ThermoFischer	Cat# C34564
Foxp3 / Transcription Factor Staining Buffer Set	Invitrogen eBioscience	Cat# 00-5523-00
Live Dead fixable blue/UV	ThermoFischer	Cat# L34961
UltraComp eBeads	Invitrogen	Cat# 01-2222-42
RNeasy	Qiagen	Cat# 74106
RevertAid First Strand cDNA Synthesis Kit	ThermoFisher	Cat# K1622
Fast SYBR green master mix	Applied Biosystems	Cat# 4385612
Chemicals, peptides, and recombinant proteins		
rmEGF	R&D	Cat# 236-EG-01M
rhIL-2 (CF)	BioLegend	Cat# 589104
rmIL-7	BioLegend	Cat# 577806
rmIL-33	BioLegend	Cat# 580502
rmFGF10	PeproTech	Cat# 100-26-50ug
rmFGF7	R&D	Cat# 251-KG-010/CF
rhIL-23	ThermoFischer	Cat# PHC9324-10
rhIL-18	BioLegend	Cat# 592102
rhIL-33	BioLegend	Cat# 581802
Advanced DMEM/F12	Gibco	Cat# 12634-010
Glutamax	Gibco	Cat# 1932118
Antibiotic-Antimycotic	Gibco	Cat# 15240096
HEPES 1M	Gibco	Cat# 2259252
2-Mercaptoethanol (βME) 50mM	Gibco	Cat# 31350-010
PFA 16%	Pierce	Cat# 28906
heat inactivated qualified FBS/FCS	Gibco	Cat# 26140087
N-Acetylcysteine	Sigma-Aldrich	Cat# A9165
Matrigel	Corning	Cat# 356231
Vitronectin XF	Stem Cell Technologies	Cat# 07180
Essential 8™ Medium	Gibco	Cat# A1517001
Versene	Gibco	Cat# 15040033
Y-27632 dihydrochloride (Rho-K inhibitor)	Tocris	Cat# 1254
Recombinant Human/Mouse/Rat Activin A Protein	Bio-Techne	Cat# 338-AC-050
Recombinant Human FGF-4 (aa 71-206)	Bio-Techne	Cat# 7460-F4-025
CHIR-99021 (CHIR)	TOCRIS	Cat# 4423
B27 supplement	Gibco	Cat# 17504-044
Ficoll 400	Sigma	Cat# 26873-85-8
PerColl	cytiva	Cat# 17089101
TrypLE-phenol free	Gibco	Cat# 12604-021
ACK lysis buffer	Lonza	Cat# BP10-548E
Succinate	Sigma	Cat# S3674-100G
FITC – 4kDa	Sigma	Cat# FD4-100MG
Brefeldin A	Invitrogen	Cat# 00-4506-51
Monensin	Sigma	Cat# M5273
PMA	Sigma-Aldrich	Cat# P1585
Ionomycin	Sigma-Aldrich	Cat# I0634
Texas Red™-X Phalloidin	ThermoFischer	Cat# T7471
DAPI	ThermoFischer	Cat# 62248
Hoechst	ThermoFischer	Cat# 62249

**Experimental models: Cell lines**		

KUTE-4, BOBC, and FS13B human induced pluripotent stem cell lines	Feeder-free hiPSC derived from skin tissue via CtyoTune 2 in 2015 as part of the HipSci consortium phenotyping project (Kilpinen et al.,(2017). The cell lines are banked at ECACC, disease status of the female anonymous donors was normal, and all details and pluripotency scores can be found at http://www.hipsci.org/lines/#/lines/HPSI0714i-kute_4. The authors acknowledge the Wellcome Trust Sanger Institute and the Wellcome Trust and MRC Cambridge Stem Cell Institute as the sources of human iPS cell lines. Mycoplasma testing was performed by the King’s College London Centre for Stem Cells and Regenerative Medicine on a monthly basis.	Addgene AAV5; 44361-AAV5
Cultrex HA-RSpondin1-Fc HEK293T Cells	Cell line was used to harvest conditioned RSpondin1 supernatant, the cell line and Materials Transfer Agreement was provided by the Board of Trustees of the Lelands Stanford Junior University (Calvin Kuo, MD,PhD, Stanford University)	N/A
HEK-293T-mNoggin-Fc Cells	Cell line was used to harvest conditioned Noggin supernatant, cell line acquired through Materials Transfer Agreement with the Hubrecth Institute, Uppsalalaan8, 3584 CT Utrecht, The Netherlands, and is based on the publication by Farin, Van Es, and Clevers Gastroenterology (2012).	N/A
Human KUTE-4, BOBC, and FS13B human induced pluripotent stem cell lines	Feeder-free hiPSC derived from skin tissue via CytoTune 2 in 2015 as part of the HipSci consortium phenotyping project (Kilpinen et al. (2017). The cell lines are banked at ECACC, disease status of the female anonymous donors was normal, and all details and pluripotency scores can be found at http://www.hipsci.org/lines/#/lines/HPSI0714i-kute_4. The authors acknowledge the Wellcome Trust Sanger Institute and the Wellcome Trust and MRC Cambridge Stem Cell Institute as the sources of human iPS cell lines. Mycoplasma testing was performed by the King’s College London Centre for Stem Cells and Regenerative Medicine on a monthly basis.	N/A

**Biological samples**		

PBMC Leukocyte cones	National Blood Transfusion Service (NHS-BT blood and transplantation, Tooting, London, UK). Human studies were conducted in accordance with the Helsinki Declaration and approved by the Institutional Review Board of Guy’s Hospital.	N/A

**Experimental models: Animal strains**		

Rorc(γt)-GfgTG, C75Bl/6J reporter mice	A generous gift by Gérard Eberl. (Lochner et al., 2008)	
C75Bl/6J	Charles river	
Germ Free C75Bl/6J	Biological Research Facility, St George’s University of London.(from M. A. Curtis)	
CD45.1, C75Bl/6J	Charles River	
Tbx21^−/−^, C75Bl/6J	Charles River	
Tbet-YFP, C75Bl/6J	(Schroeder et al., 2020)	
Tbet-AmCyan, C75Bl/6J	A generous gift by J Zhu.(Yu et al., 2015)	

**Deposited data**		

Raw RNA-sequencing data of SIO cultured with or with ILC and ILC medium.	This paper; Figure 3L	NCBI Bioproject: PRJNA851572. https://www.ncbi.nlm.nih.gov/bioproject/PRJNA851572

**Oligonucleotides**		

*Il25* FW: TGGCTGAAGT GGAGCTCTGCAT REV: CCCGATTCAAGTCCCTGTCCAA	Invitrogen	Custom
*Il33* FW: CTACTGCATGAGACT CCGTTCTG REV: AGAA TCCCGTGGATAGGCAGAG	Invitrogen	Custom
*Hprt1* FW: CTGGTGAAAAGGA CCTCTCGAAG REV: CCAGTTTC ACTAATGACACAAACG	Invitrogen	Custom
*Gapdh* FW: CATCACTGCCACCC AGAAGACTG REV: ATGCCAG TGAGCTTCCCGTTCAG	Invitrogen	Custom

**Software and algorithms**		

FIJI (Fiji Is Just ImageJ)	(Schindelin et al., 2012)	https://imagej.net/software/fiji
FlowJo	BD Life Sciences	FlowJo™ Software (for Mac) Version 10.4.1.https://www.flowjo.com/
Graphpad Prism 9	Dotmatics	www.graphpad.com
Microsoft Office 365 Excel 16.16.20	Microsoft	www.microsoft.com
QIAGEN Ingenuity Pathway Analysis (IPA)	(Krämer et al., 2014)	https://digitalinsights.qiagen.com/products-overview/discovery-insights-portfolio/analysis-and-visualization/qiagen-ipa/

**Other**		

ViewPlate-96 Black, Optically Clear Bottom, Tissue Culture Treated, Sterile	PerkinElmer	6005182

### Resource availability

#### Lead contact

• Further information and requests for resources and reagents should be directed to and will be fulfilled by the lead contact, Joana F Neves (joana.pereira_das_neves@kcl.ac.uk).

#### Materials availability

• This study did not generate new unique reagents.

### Experimental model and subject details

#### Animals

Rorc(γt)-GfgTG, C75Bl/6J reporter mice (gift by Gérard Eberl), SPF C75Bl/6J, CD45.1, C75Bl/6J, Tbx21^−/−^, C75Bl/6J, Tbet-YFP, C75Bl/6J, Tbet-AmCyan, C75Bl/6J≤ (gift by J Zhu), Germ Free C75Bl/6J 6–12 weeks females were used.

All animals were culled by cervical dislocation according to standard ethical procedure, conducted by trained individuals. Slicing of the femoral artery or decapitation was conducted (as appropriate to the protocol in hand) as confirmatory assessments of death, prior to organ and tissue harvest.

Animals were housed under specific-pathogen-free conditions (unless stated otherwise) at accredited Charles River and King’s College London animal units in accordance with the UK Animals (Scientific Procedures) Act 1986 (UK Home Office Project License (PPL:70/7869 to September 2018; P9720273E from September 2018).

Germ-free animals were maintained by MAC in IVCs inside isolators under axenic conditions at the Biological Research Facility, St George’s University of London in accordance with the UK Animals (Scientific Procedures) Act 1986 (UK Home Office Project License P5DB2B893).

#### Cell lines

KUTE-4 (Kilpinen et al., 2017) FS13B and BOBC (Yusa et al., 2011) human induced pluripotent feeder free stem cell lines were grown at 37°C, 5% CO2. Mycoplasma testing was performed by the King’s College London Centre for Stem Cells and Regenerative Medicine on a monthly basis.

Cultrex HA-RSpondin1-Fc HEK293T Cells and HEK-293T-mNoggin-Fc Cells were used to harvest conditioned RSpondin1 supernatant and conditioned Noggin supernatant respectively.

### Method details

#### Murine organoids isolation

Murine epithelial small intestines (ileum) were isolated from 6-8 week female C57BL/6 mice following established protocols (Sato et al., 2011). Intact intestinal stem cell crypts were resuspended in 25 μL ice-cold Matrigel bubbles and gently plated onto prewarmed tissue culture plates. Resulting SIO were passaged using a bent pipette tip to mechanically disrupt structures into individual whole crypt buds every 5–7 days. For expansion, SIO were cultured in basal media supplemented with R-Spondin1 (50 μL supernatant/mL or 1 mg/mL), Noggin (50 μL supernatant/mL or 100 μg/mL), and rm-EGF 50 μg/mL), referred to as SIO medium.

Murine lung organoids were isolated from distal lung tips following established protocols (McQualter et al., 2010). Tissue was cut into small 5–20 mm^2^ pieces, rinsed in PBS, then digested using 1.5 mg/mL Dispase II, 0.5 mg/mL Collagenase, and 10 μg/mL DNAse in 10 mL PBS with 2% FCS for 1 h at 37°C on a shaker set to 100–250 RPM, with a 15 s vortex every 20 min. Samples were then allowed to settle, and a cloudy fraction enriched for fibroblasts and immune cells was discarded. The remaining larger chunks were then incubated in EDTA and HEPES for another hour, before being resuspended in Matrigel, and cultured in SIO media supplemented with FGF10 (500 ng/mL) and 5 μM CHIR (LO expansion media) and 1 μM RhoK-inhibitor for 4 days. Once luminal structures formed, RhoK-inhibitor was withdrawn, and cultures were expanded for a minimum of three weeks to enrich for alveolar basal stem cells. These heterogeneous organoid cultures were then FACS purified to isolate Live, CD45-, EpCAM^high^ epithelial cells, which were expanded as epithelial only structures for 4 weeks in LO expansion media. CHIR and FGF10 were withdrawn and LO transferred to SIO media on the day prior to establishing co-cultures, enabling lung epithelial differentiation and to maintain constant culture conditions between tissues.

#### Human organoid differentiations and stromal depletion

KUTE-4 (Leha et al., 2016) and FS13B human iPSCs previously generated using established protocols (Kilpinen et al., 2017; Yusa et al., 2011). Human iPSCs were maintained on Vitronectin (StemCell Technologies) coated tissue culture plates in E8 media. Media was changed every day and pockets of differentiation manually removed, and round pluripotent colonies were passaged as disrupted clusters without RhoK inhibitor every 4–6 days using Versene (GIBCO).

HIO were derived following previously established protocols (McCracken et al., 2011), with substitution of CHIR99021 for recombinant Wnt3a (Tsai et al., 2017). HIO were further matured through addition of 20 ng/mL IL-2 to the expansion media (Jung et al., 2018). HIO were passaged and reseeded in Matrigel as whole structures every 10–14 days. Organoids were matured for 4–8 weeks in expansion media before being used in co-culture experiments.

HLO were differentiated using definitive endoderm specification (Kilpinen et al., 2017) and subsequent lung airway epithelial differentiation (Konishi et al., 2016). Lung progenitors were sorted and seeded in 3D domes made with a 1:1 mix of medium supplemented with CHIR 3uM, Y-27632 10uM and FGF10 100ng/mL and Growth Factor Reduced Matrigel (356,231, Corning). The domes were then topped up with the same medium. Maturation was promoted through addition of FGF7 and FGF10 (de Carvalho et al., 2019). HLO were dissociated as single cells and matured in Rho-K inhibitor and CHIR for two weeks, then matured in complete media containing FGF10, FGF7, and IL-2 for an additional two weeks prior to use in co-cultures.

For stromal depletions, HIO/HLO were digested with 1mL 0.1 mg/mL Collagenase in Advanced DMEM12 for 15 min at 37°C, then mechanically disrupted until a cloudy, stromal-enriched fraction appeared in the supernatant. Epithelial structures were allowed to settle to the base of a 15 mL falcon tube, and the mesenchyme-rich fraction was carefully separated into the HIO-STRO fraction. This step was repeated 2–5 times or until the epithelial-enriched fraction remained clear even after disruption.

#### Murine ILCP and ILC isolation

ILCP were isolated from 6-8 week C57BL/6 female murine femur and tibia bone marrow (BM) following established protocols (Gronke et al., 2017). Soft tissue was physically removed from bones, which were subsequently sterilised in 70% ethanol for 2 min and rinsed in ice cold PBS. The ends of the bones were cut off with dissecting scissors and flushed with PBS using a 27-gage needle. Marrow was triturated, transferred to a 50 mL falcon tube, and centrifuged at 1500 RPM/500 G for 3 min. After removal of supernatant, red blood cells were depleted using 2 mL standard ACK lysis buffer for 2 min at room temperature. Mature primary tissue ILC were isolated from 6-8 week C57BL/6 female murine small intestine following established protocols (Jowett et al., 2021).

The reaction was quenched with 30 mL PBS and centrifuged at 500 x G for 3 min. The remaining pellet was resuspended in PBS supplemented with 2% FCS, 0.1 M EDTA, and 1mM HEPES (FACS buffer), and strained into a flow tube through a 40 μm sterile mesh. Fc receptors were blocked using anti CD16/CD32 (2.4G2) for 10 min at 4°C. Cells were stained with primary conjugated antibodies in the dark at 4°C for 30 min 5 million cells in 100 μL FACS buffer, rinsed in FACS buffer, centrifuged, and resuspended in 200–300 μL FACS buffer with 0.1μg/mL DAPI (4′,6-diamidino-2-phenylindole; Sigma) for dead cell exclusion. Unstained cells and UltraComp beads (eBiosciences) were used for unstained and single colour controls to calculate compensation. Fluorescence minus one (FMO) were used for CD127 (IL 7Rα) and Lineage + DAPI ChiLP were isolated by Fluorescence-activated cell sorting (FACS) on an ARIA-III (BD Biosciences) using DIVA software, with assistance from the Guy’s King’s St Thomas (GSST) Biomedical Research Council (BRC) flow core staff.

#### Human ILCP isolation

Systemic ILCP were isolated from leukocyte cones (NHS-BT) following established protocols (Lim et al., 2017). Briefly, lymphocytes were purified from cones using FICOLL density gradient separation. Single cells were Fc blocked, rinsed with PBS for fixable Live/Dead staining, stained using the ILCP panel, and sorted on a FACSARIA-III (BD biosciences) to be used in downstream co-cultures.

#### ILCP co-cultures with organoids

Murine co-cultures were established following established protocols (Read et al., 2022). Of note, SIO/LO were consistently passaged 2 days prior to establishing co-cultures, to ensure that differentiated cells were consistently present, and to ensure that structures were large enough to pellet upon centrifugation. Unlike passaging protocols, SIO and LO were not mechanically disrupted, but seeded into co-culture as intact crypts. Approximately 50–100 murine SIO were seeded with 500–1000 ILCP for each condition, maintain an approximate SIO 1:10 ILCP ratio. For co-cultures, SIO expansion media was supplemented with 50 mM β-mercaptoethanol (R&D), 20 ng/mL rhIL-2 (Sigma), and 20 ng/mL rmIL-7 (R&D), referred to as ILC media.

Human co-cultures were established by adapting established protocols (Jowett et al., 2021). Approximately 20–50 HIO/HLO epithelial structures were seeded with 200–500 ILCP, maintaining an approximate SD-O 1:10 ILCP ratio. Critically, stromal depletion and enrichment were performed not only during routine passaging but were performed rigorously on the day of establishing co-culture to maximise compartmental separation. ILC media was added as in the murine samples.

For co-cultures, half media changes were performed with 2X concentrated media containing twice the amount of recombinant growth factors were performed every 1–2 days, (50% media out, replenished with 60% of the remaining volume to account for evaporation). This allowed for SIO/ILCP conditioned media to remain in the wells without disrupting ILC-epithelial interactions, while supplementing with sufficient resources to promote optimal viability of organoids. No small molecules, FGF7, or FGF10 were supplemented to lung organoid cultures to maintain consistency between SIO/LO and HIO/HLO conditions.

For transwell/TW experiments, permeable inserts separated the organoid fraction (top) and the ILCP fraction (bottom), with both resuspended in 25 μL Matrigel (Falcon 24 well (Corning), 1.6 × 10⁶ pores per cm^2^).

In inter-organ swapping experiments, ILCP + organoid co-cultures were dissociated with TryPLE on day 7 and each condition split evenly into two tubes. One-half was stained and sorted for DAPI- EpCAM-, CD45+, Lineage-whole populations by FACS, with the other half being used for intracellular flow cytometry analysis (using the FOXP3 staining kit). This prevented extra-cellular epitopes like Klrg1 from being blocked for secondary analysis on day 14, and ensured that no lingering fluorescence impacted downstream analysis. The organoid-matured ILC populations yielded by FACS were then re-seeded with the same or the opposite organoid cultures in fresh Matrigel, and the protocol was restarted as on day 1.

For regular analysis, day 7 or day 14 (swapped) co-cultures were either fixed in 4% PFA for immunocytochemistry, or rinsed with PBS and dissociated with TrypLE (Gibco) and DNAse (due to potential dead epithelial cells) for 20 min to obtain single-cell suspension to be analysed by flow, or to FACS purify individual populations directly into cooled lysis buffer (RLT) supplemented with 10 μL/mL βME for downstream RT-qPCR. Any suspension containing single epithelial cells were maintained in 2% FCS with 0.1 mM EDTA and 1mM HEPES to reduce cell clumping. Stimulation with PMA/Ionomycin for intracellular cytokine staining was performed for 4h at 37°C 5% CO_2,_ and samples were gently vortexed or shaken every 30–60min to ensure even distribution. Monensin and Brefeldin A (golgi inhibitors) were added for the entire duration of the stimulation, and were supplemented in the TryPLE, extra-cellular staining buffers, and the fixation buffer of the FoxP3 staining kit to ensure cytokine staining was not lost. For additional subset specific cytokine stimulation IL-23 or IL-18 were added to the PMA/Ionomycin/Brefeldin A/Monensin stimulation cocktail.

#### Co-culture analysis

##### Flow cytometry

Flow cytometry data was acquired on a Fortessa II (BD Biosciences) using DIVA software and analysed using FlowJo 10.4.1. BD compensation beads were used to acquire single colour controls for accurate compensation of multi-colour panels, and fluorescence minus one (FMO) controls used when appropriate.

##### RT-qPCR

RNA was extracted using the RNAeasy micro kit (Qiagen), with 10 μL/mL β-ME supplemented to the RLT lysis buffer to mitigate degradation in RNAse-rich epithelial tissues. Next, cDNA reverse transcription was performed following manufacturer’s protocols with the RevertAid synthesis kit (ThermoFischer), using 0.5 μL random primer and 0.5 μL Oligo dTTT primer per 10μL reaction. RT-qPCR were with primers ordered from Invitrogen with SYBR (Applied Bioscences) and run on a BioRad Real Time CFX384 Touch with CFX Maestro software, and resulting data were processed and normalised in Microsoft excel.

##### Confocal and live imaging

Live imaging was performed overnight on co-cultures 1 day post-seeding for Cell Trace FarRed experiments where ILCP were labeled prior to co-culture, or on day 4 for ILCP cultures with Rorc^eGFP^ animals. Images were taken using a NIKON A1R inverted confocal microscope with incubation capabilities, and using phenol free media. Co-cultures were then fixed in 4% PFA for 5–15 min at room temperature within 3D Matrigel bubbles to retain relative ILC-organoid localisation. Samples were either stained as whole organoids, having been plated on glass-bottom 96-well plates (perkin elmer) to allow for minimal co-culture disruption. Supernatant was manually removed, and wells were never allowed to dry. Samples were permeabilised using 0.05% Triton-X, stained in primary antibody overnight at 4C, and stained in secondary antibody and Hoechst for 1h at room temperature (RT). Images were acquired on a Leica SP8 confocal microscope using LAX software, and resulting images were processed using FIJI (ImageJ).

#### RNA-sequencing of murine SIO

Lymphocyte precursors (Lin^−^, Cd127^+^, α4β7-, Ftl3^+^) where harvested from the BM and sorted as described above and cultured with SIO in the presence of 50 mM β-mercaptoethanol (R&D), 20 ng/mL rhIL-2 (Sigma), 20 ng/mL rmIL-7 (R&D) and 20 ng/mL Flt3-ligand (R&D) for 7-days. EpCAM^+^Cd45^-^ were sorted into lysis buffer and RNA was extracted as described above. The library was prepared using SMARTer Stranded Total RNA Seq kit - pico input mammalian and sequenced using HiSeq 2500 at the King’s College London Genomics Centre, where basic alignment and quality control were also performed. Normalised count values were represented as (logX+1,2) in Excel 16.16.20, and represented as a heatmap in GraphPad Prism 8.2.1.

### Quantification and statistical analysis

Data was analysed using Microsoft Office 365 Excel 16.16.20 and GraphPad Prism 8.2.1. Meta-analysis of public deposited RNA-sequencing was performed by normalising raw FPKM values to the Geometric mean of housekeeping genes *Actb/ACTB*, *Hprt1/HPRT1*, and *Gapdh/GAPDH*, and the (logX+1, 2) of these values was analysed with multiple row t-tests. Log-q values were visualised as Volcano plots in GraphPad Prism 8, heatmaps were produced using heatmapper.ca/expression/, applying clustering to rows and columns (applying clustering dendrograms to columns) using centroid linkage and Eucledian distance measurement. Gene pathway analysis was performed using QIAGEN Ingenuity Pathway Analysis (IPA).

## Data Availability

• RNA-seq data have been deposited at GEO and are publicly available as of the date of publication. Accession number is listed in the key resources table. Microscopy data reported in this paper will be shared by the lead contact upon request. • No original code was written for this publication.

## Supplemental information

Document S1. Figures S1–S7

Data S1. Analyzed RNA sequencing data comparing IECs from SIOs cultured with ILCs versus SIOs cultured with ILC medium

Document S2. Article plus supplemental information
