# Supplementary material for: Organoids capture tissue-specific innate lymphoid cell development in mice and humans
Source: Cell Rep. 2022 Aug 30;40(9):111281. doi: 10.1016/j.celrep.2022.111281 (PMC9638027; doi:10.1016/j.celrep.2022.111281)
Supplement: Document S1. Figures S1–S7 [file mmc1.pdf]

Cell Reports, Volume *40*

Supplemental information

Organoids capture tissue-specific innate lymphoid cell development in mice and humans

Geraldine M. Jowett, Emily Read, Luke B. Roberts, Diana Coman, Marta Vilà González, Tomasz Zabinski, Umar Niazi, Rita Reis, Tung-Jui Trieu, Davide Danovi, Eileen Gentleman, Ludovic Vallier, Michael A. Curtis, Graham M. Lord, and Joana F. Neves

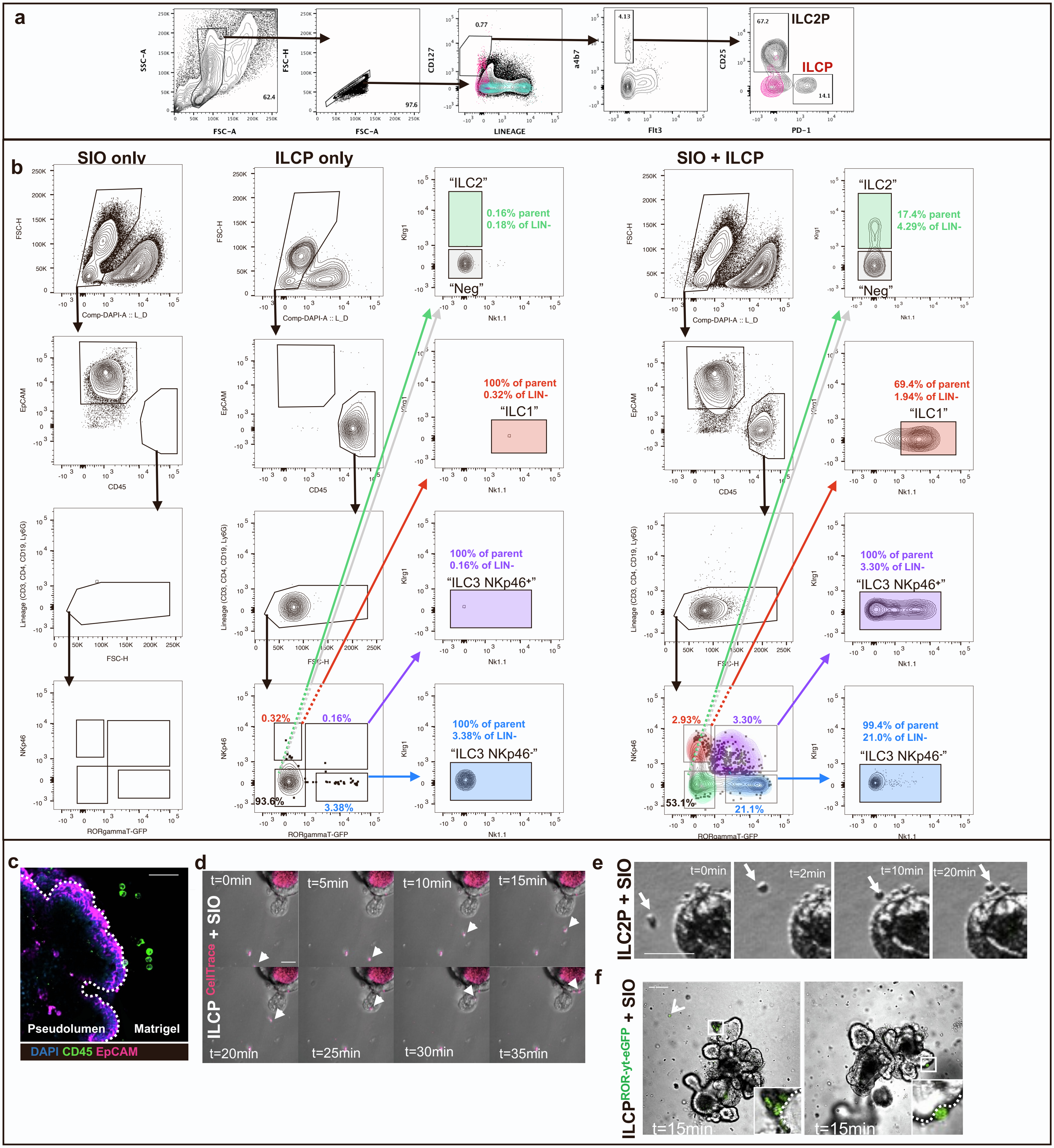

Supplementary figure 1 relating to Figure 1

a) FACS gating strategy to isolate innate lymphoid precursors (ILCP) from murine bone marrow, with appropriate FMO controls overlayed in cyan and magenta. **b)** FACS gating strategy to isolate SIO, SIO+ILCP, or ILCP only after 7 day culture in 3D Matrigel. This method is additionally used to classify group 1 (red), group 2 (green), and group 3 (NKp46$^+$ (purple) NKp46$^-$ (blue) in DAPI$^-$, EpCAM$^-$, CD45$^+$, Lineage$^-$ putative ILC based on unbiased, unstimulated live expression of extra-cellular markers. **c)** Representative confocal cross-section of EpCAM$^+$ SIO (magenta, DAPI blue) and ILCP-derived CD45$^+$ ILC (green). White dotted line delineates the apical pseudolumen, and ILCP reside the basal lamina propria (Matrigel). **d)** Stills taken from live imaging of ILCP labeled with CellTrace-FarRed, with white arrow delineating movement of a labeled ILC toward the organoid (image every 5min). **e)** Phase contrast live images of ILC2P co-cultured with SIO showing close interaction of an ILC (white arrow) with and intestinal organoid. **f)** Frames from SIO co-cultures with ROR-yt^{eGFP} labeling ILCP-derived ILC3 after 4-day co-culture. Scale bars 50μm.

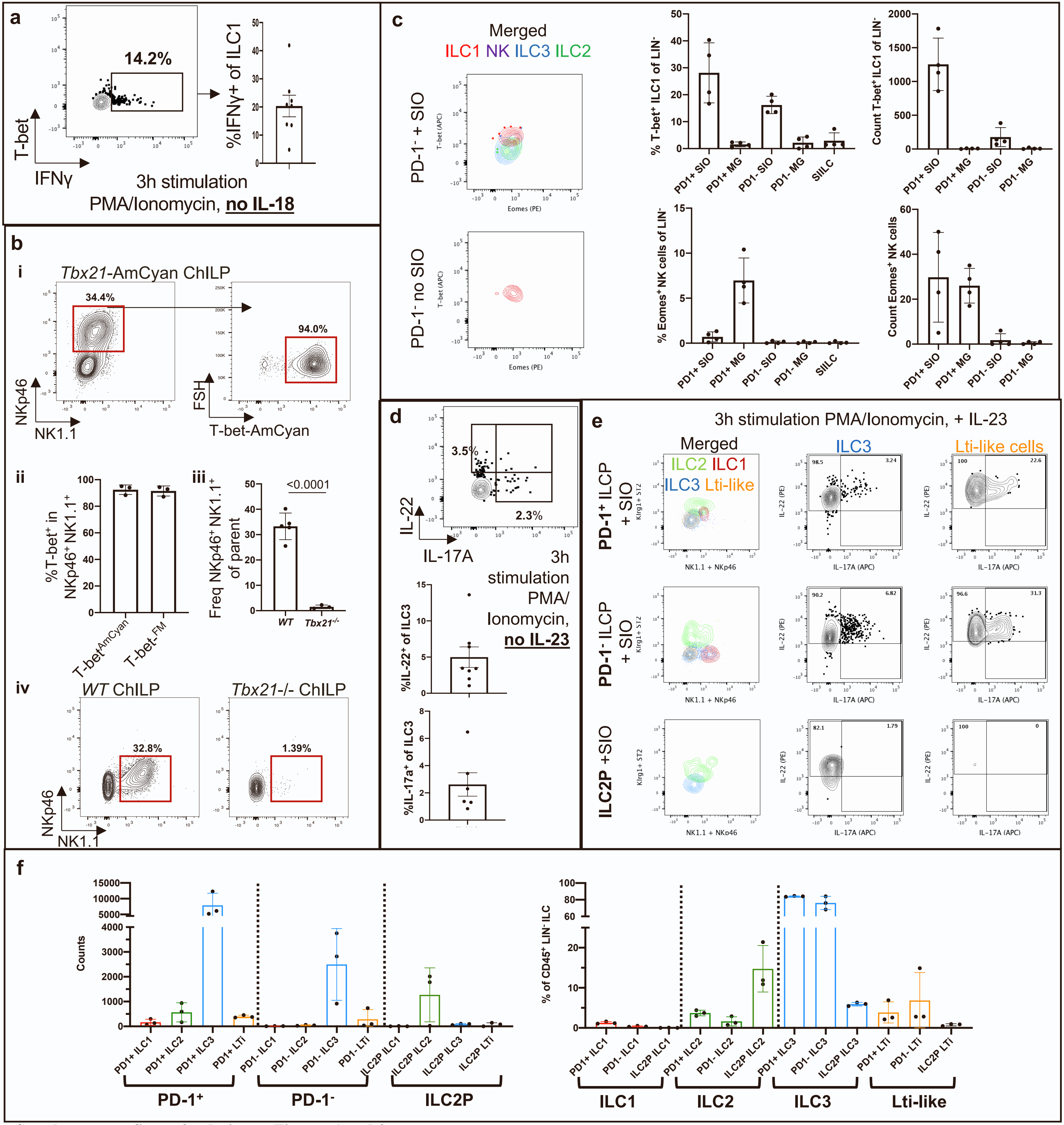

Supplementary figure 2 relating to Figures 1 and 2

a) Representative flow plot of IFN-γ expression after 4h PMA/Ionomycin stimulation in T-bet^{+} ILC1 (RORγt^{-} NKp46^{+} NK1.1^{+}, Klrg1^{-}) with corresponding quantification (N= 8 mice across at least two experiments). **b) i)** Representative flow plot of Tbet-AmCyan reporter mouse (Tbet^{AmCyan})-derived ILCP cultured with SIO, pre-gated on EpCAM^{-} CD45^{+} Lin^{-} Klrg1^{-} RORγt^{-}. **ii)** Expression of T-bet from T-bet^{Amcyan} and T-bet fate YPF mapper (Tbet-FM) in putative NK1.1^{+} NKp46^{+} ILC1. **iii)** Quantification of EpCAM^{-} CD45^{+} Klrg1^{-} NKp46^{+} NK1.1^{+} ILC1 derived from 7 day co-cultures of SIO with ILCP derived from Wildtype (WT) control or *Tbx21*-/- knockout mice (unpaired student t-test). **iv)** Representative flow plots relating to **(iii)**. **c)** Representative flow plots overlaying putative ILC1, NK cells, ILC3, and ILC2 derived from co-culture of PD-1^{-} ILCP with or without SIO with corresponding quantifications. All error bars S.E.M.. **d)** Representative flow plot of IL-22 and IL-17A expression in ILCP-derived ROR-γt+ group 3 ILC after 3h stimulation with PMA/Ionomcyin and IL-23 with corresponding quantification(N=8 mice). **e)** Representative flow plots overlaying putative ILC2, ILC1, ILC3, and Lti-like cells after SIO co-culture with PD-1^{+}ILCP, PD-1^{-} ILCP, and CD25^{+} ILC2P **f)** with corresponding quantification of count and frequency organised by seeded precursor or by resulting ILC populations (N=3 mice). All error bars S.E.M..

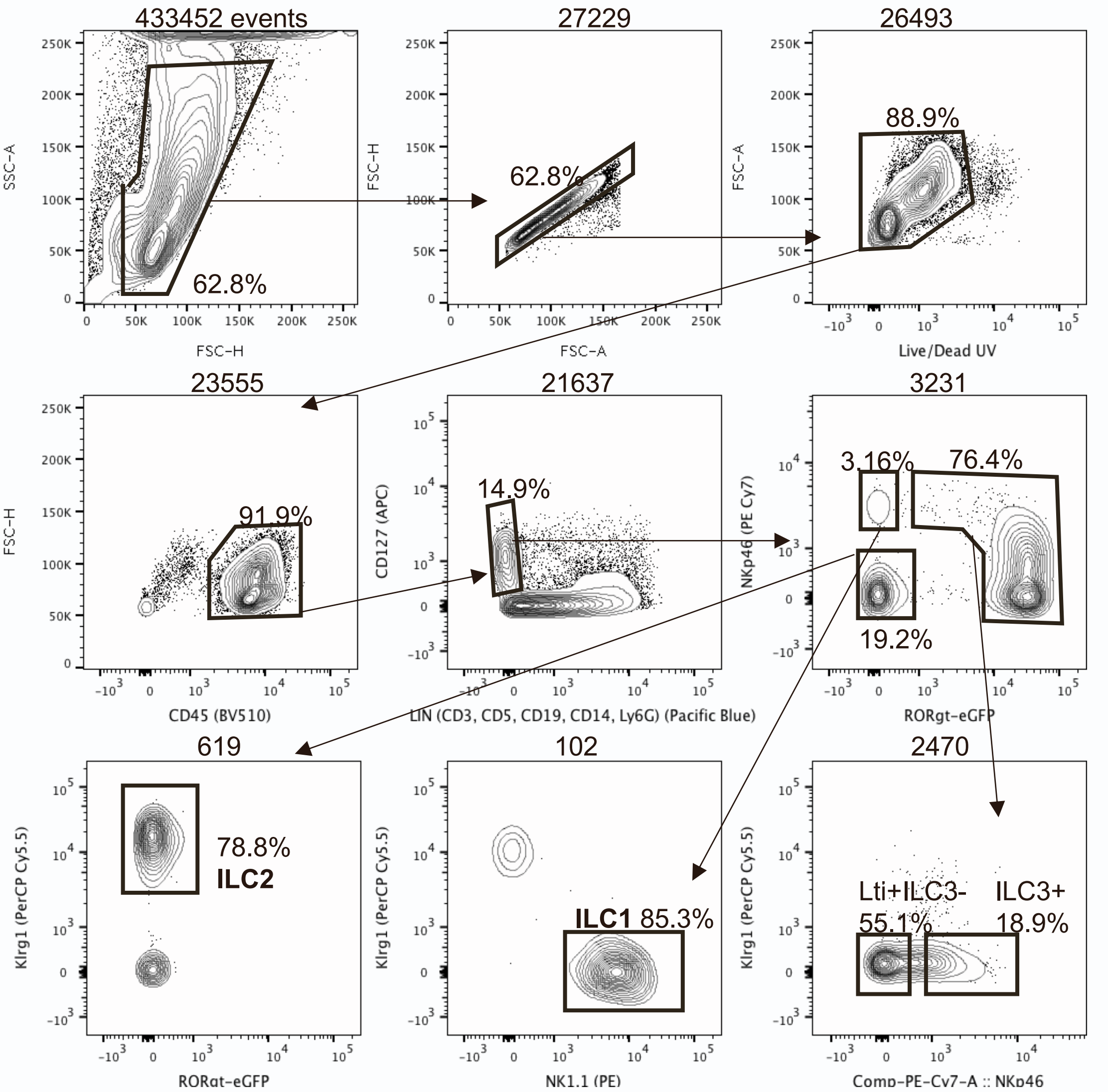

Supplementary figure 3 relating to Figure 3

Representative flow plots of gating strategy for identifying relative frequency of group 1, group 2, and NKp46$^{+/-}$ group 3 ILC from primary murine small intestine lamina propria (with Peyer's Patches physically removed prior to lymphocyte isolation). Absolute cell number within each gate listed above each plot.

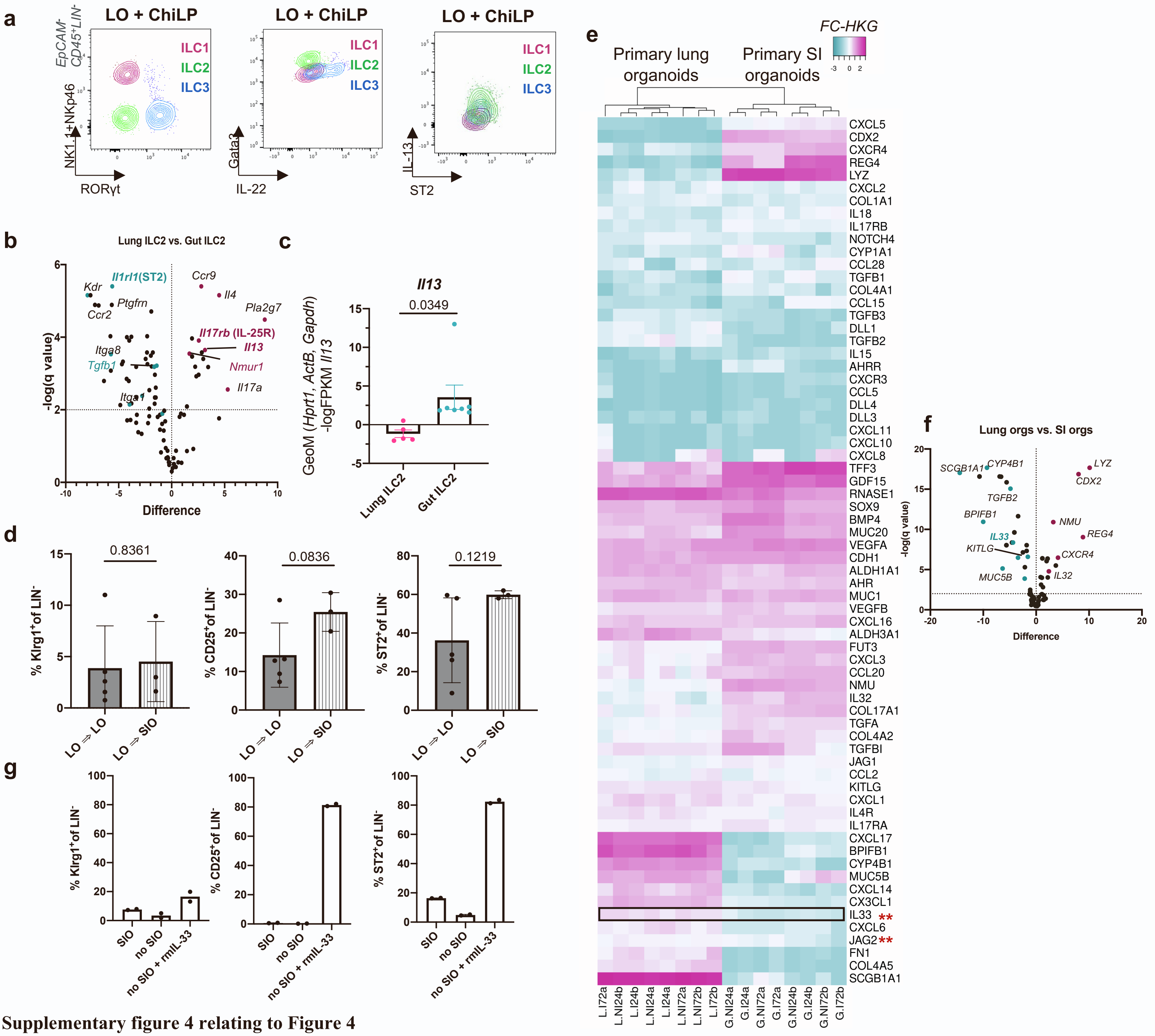

Supplementary figure 4 relating to Figure 4

a) Representative flow plots of overlaying putative group 1, group 2, and group 3 ILC after 7 day co-culture with murine lung organoids (LO). **b)** Volcano plot of differential gene expression in murine primary lung (left) and gut (right) ILC2, adapted from primary RNA-sequencing data from GSE117568 (Ricardo-Gonzalez et al., 2018a), showing the log(q-value) raw FPKM values were normalised to the geometric mean of housekeeping genes *Hprt1, Actb, and Gapdh* (q value: discovery (above the horizontal line) determined using the Two-stage linear step-up procedure of Benjamini, Krieger and Yekutieli, with Q = 1%. Each row was analyzed individually, without assuming a consistent SD.). **c)** RNA levels of *Il13* in gut and lung highlighted and represented as a bar blot, showing relative decrease in *Il13* expression in pulmonary ILC2. **d)**. Frequency of Klrg1, CD25, and ST2 positive cells among putative ILC2 derived from 7 day LO co-culture after FACS isolation from co-cultures and reseeding in Matrigel with LO or swapped to SIO culture for an additional 7 days. (N=3). **e)** Eucledian heatmap (average linkage) of genes of interest expressed in human gut organoids (G;N=2) and lung organoids (L; N=2), cultured for 24h or 72h, not infected (NI) or infected (I) with *Cryptopsporidium,* representative of N=3 experiments. Values represent [log (X+1,2)] fold change (FC) relative to housekeeping genes (HKGs) *ACTB, GAPDH*, and *HPRT1* (magenta: greater than HKG, cyan: lower than HKG). Black box highlights significantly differential expression of gene of interest *IL33* (Two-stage linear step-up of Benjamini, Krieger, and Yekultieli, with Q=1%, p <0.000001; q<0.000001). (Accession: GSE112991) of human primary small intestine and lung epithelial organoids, sequenced after 24h or 72h culture with (infected - I) or without (not infected - NI) microinjected *Cryptosporidium* infection (Heo and Dutta et al., 2018). Ingenuity Pathway Analysis (IPA) was used to annotate these datasets, isolating basally presented or secreted ligands, as well as quality control genes (e.g. CDX2 in intestinal epithelium) **f)** Volcano plot of data represented in (a), highlighting genes with statistically significant differential expression. **g)** Frequency of Klrg1, CD25, and ST2 positive cells among putative ILC2 derived from 7 day SIO co-culture after FACS isolation from co-cultures and reseeding in Matrigel with or without SIO, and without SIO but with rmIL-33 supplementation for an additional 7 days (N=2 mice). Error bars S.E.M., unpaired student t-tests.

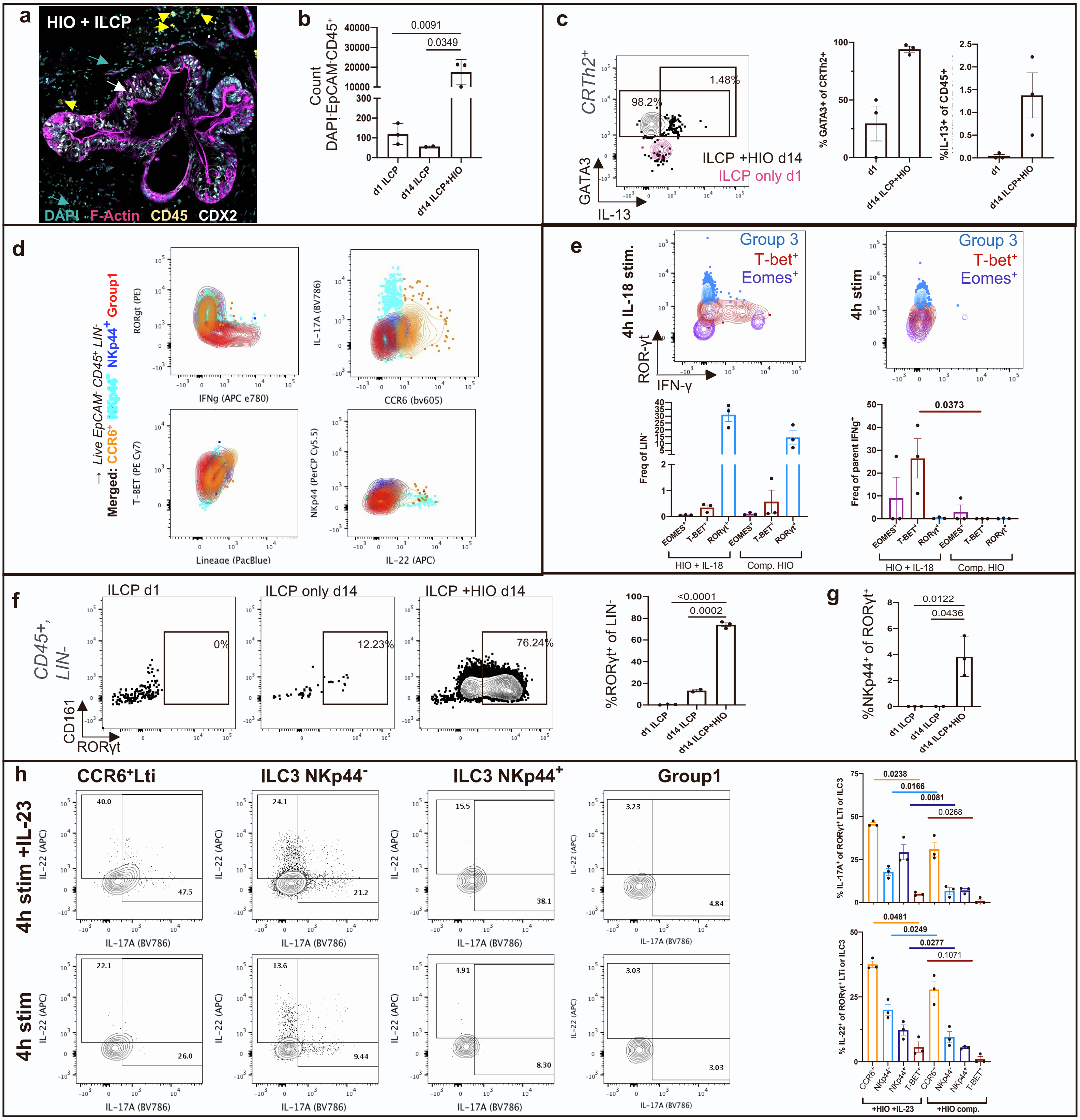

Supplementary figure 5 relating to Figure 5. a) Representative confocal image of a human intestinal organoid (HIO, apical actin ring magenta, hindgut expression of transcription factor CDX2 white, Nuclei stained with DAPI, cyan) in co-culture with ILCP (CD45, yellow arrows). Mesenchyme surrounds complete epithelial-mesenchymal HIO structures as CD45$^-$ CDX2$^-$ nuclei (blue arrows). Scale bar 50µm. **b)** Count of seeded ILCP (day 1), CD45 immune cells after 14 day co-culture with or without complete epithelial-mesenchymal HIO. **c)** Representative flow plot of GATA3 and IL-13 expression in CD45$^+$ Lineage$^-$ ROR-γt$^-$ CRTh2$^+$ ILC2, stimulated with PMA/Ionomcyin for 4h, with corresponding quantification of expression in day 1 ILCP pre-co-culture and 14 day co-culture with complete HIO (ILCP from N=3 donors). **d)** Overlays of target gene expression in putative group 1 (magenta- Live EpCAM$^-$ CD45$^+$ LIN$^-$ RORγt^{low} T-bet$^+$), and group 3 (Live EpCAM$^-$ CD45$^+$ LIN$^-$ ROR-γt$^+$) cells expressing CCR6 (orange), NKp44 (dark blue), and no NKp44 (light blue) after 4h stimulation with PMA/Ionomycin after 14 day co-culture HIO. **e)** Overlay of putative group 3, T-bet$^+$ and Eomes$^+$ group 1 ILC, showing expression of IFN-γ in these PMA/Ionomycin stimulated co-cultures with complete HIO with or without IL-18 stimulation, with corresponding quantifications. **f)** Representative flow plots CD161 and RORγt expression in Live EpCAM$^-$ CD45$^+$ LIN$^-$ HIO-derived ILC, with corresponding quantification of CD161$^+$ RORγt$^+$ cells and **g)** the frequency of NKp44$^+$ ILC within the CD161$^+$ RORγt$^+$ population. **h)** Representative flow plots of IL-22 and IL-17A expression with corresponding quantification in complete HIO-derived ILCP after 4h stimulation with PMA/Ionomycin with or without additional IL-23 stimulation. Error bars S.E.M., unpaired student t-tests, ILCP from N=3 donors.

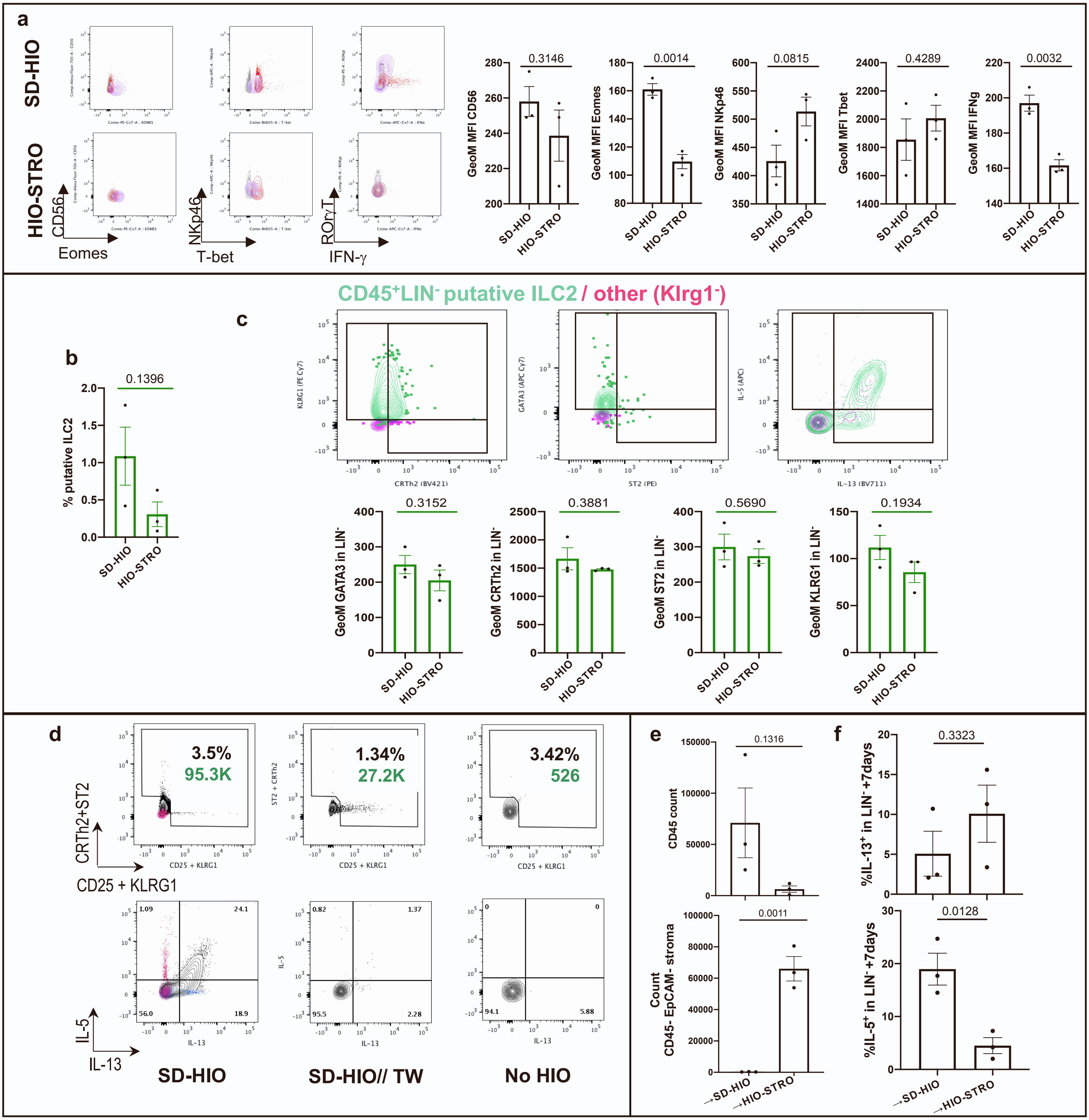

Supplementary figure 6 relating to Figure 6.

a) Representative flow plots of group 1 associated genes visualised by overlaying ROR-γt⁻ T-bet+ (red), Eomes^{+} (purple), or "other" T-bet^{-} (grey) after 14 day co-culture with SD-HIO or HIO-STRO after 4h stimulation with PMA/Ionomycin, with relevant quantification of geometric mean fluorescence intensities. Error bars S.E.M., unpaired student t-tests, ILCP from N=3 donors. **b)** Relative frequency among CD45^{+} Lineage^{-} of putative ILC2 (gating strategy in (b)), after 14 day co-culture with SD-HIO or HIO-STRO. **c)** Representative flow plots overlaying putative ILC2 (green) and other Klrg1^{-} non-ILC2 (magenta) showing expression of group 2 associated genes after 4h stimulation with PMA/Ionomycin (ILCP from N=3 donors). **d)** Representative flow plots of putative ILC2 and IL-5/IL-13 expression after 4h stimulation with PMA-Ionomycin after ILCP co-culture with SD-HIO, SD-HIO separated from ILCP by a transwell separator, or ILCP cultured in Matrigel without organoids (N=1 donor, n=2 technical replicates). **e)** Count of EpCAM^{-}CD45^{+} immune cells and EpCAM^{-}CD45^{-} fibroblasts/mesenchyme in co-cultures derived from 14-day SD-HIO co-cultures, which were then FACS purified and reseeded either with fresh SD-HIO or with HIO-STRO. **f)** Frequency of IL-13^{+} and IL-5^{+} ILC within putative ILC2 pre-matured with SD-HIO and reseeded with SD-HIO or HIO-STRO for an additional 7 days. ILCP from N=3 donors. All error bars S.E.M., unpaired student t-tests.

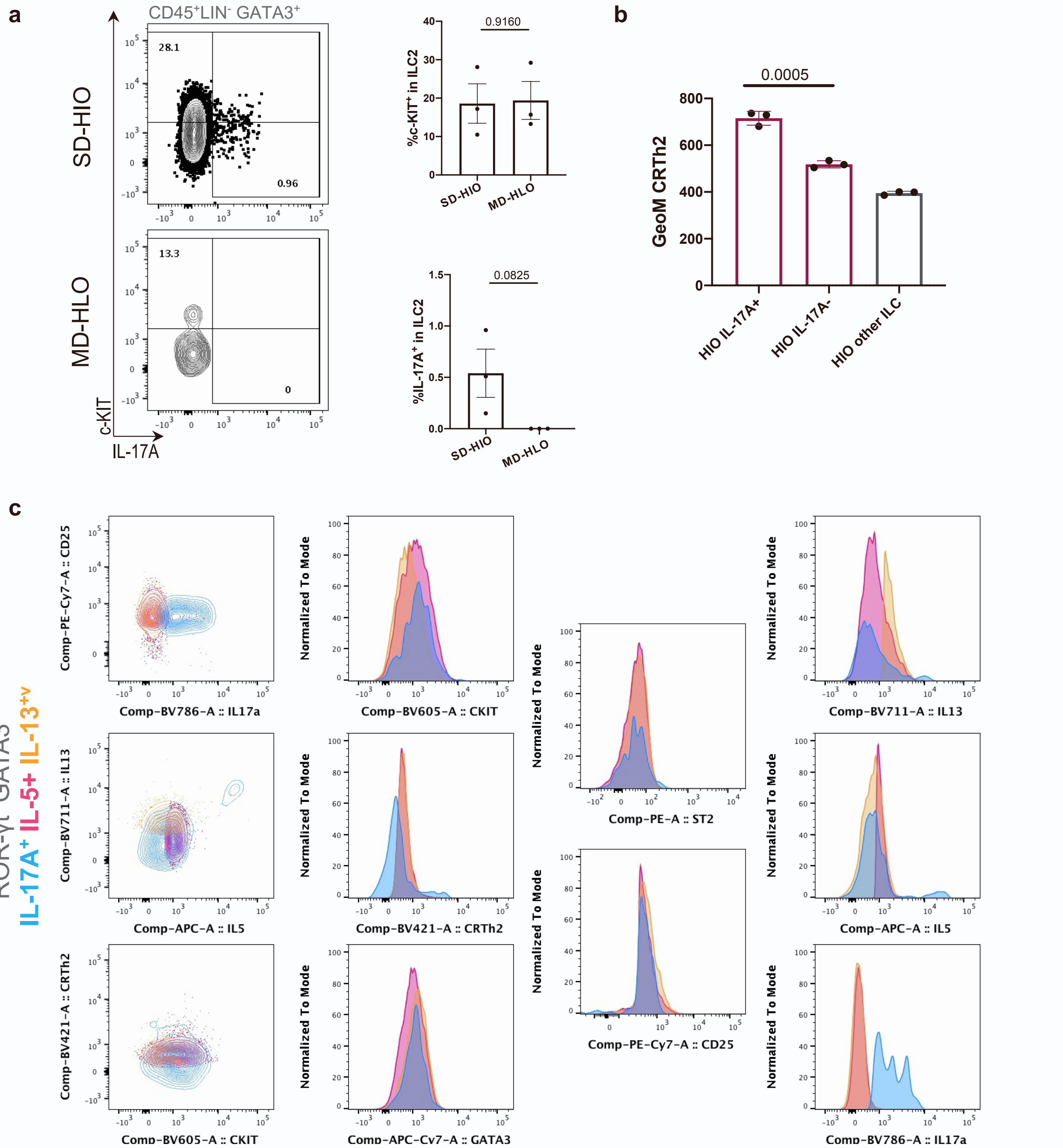

Supplementary figure 7 relating to Figure 7

a) Representative flow plots of Live, EpCAM$^-$ CD45$^+$ Lineage$^-$ RORγt$^-$, GATA3$^+$ CRTh2$^{low/+}$ expression of c-KIT and IL-17A after 4h stimulation with PMA/Ionomycin, with corresponding quantification, and **b**) MFI of CRTh2 within the IL-17A$^+$ and IL-17A$^-$, and CRTh2$^-$ HIO-derived populations. **c)** Overlay of ROR-γt$^-$ GATA3$^+$ SD-HIO co-culture derived putative ILC2 gated as IL-17A$^+$ (blue), IL-5$^+$ (magenta), or IL-13$^+$ (orange), demonstrating an increase in c-KIT and a decrease in CRTh2$^+$ but no major shift in GATA3 ST2, or CD25 expression within this ex-ILC2 ILC3-like population. ILCP from N=3 donors, error bars S.E.M., unpaired student t-test.
